# Surface‐Interaction‐Driven Polarity Switching in II–V Cd_3_P_2_ Colloidal Quantum Dots for Infrared Photodiodes

**DOI:** 10.1002/advs.202600061

**Published:** 2026-03-02

**Authors:** Ha‐Chi V. Tran, Doeun Shim, Youngsang Park, Mahnmin Choi, Hyeonjun Jeong, Guillaume Bonifas, Liyan Ouyang, Celine Nayral, Fabien Delpech, Joongoo Kang, Sohee Jeong

**Affiliations:** ^1^ Department of Energy Science (DOES) Sungkyunkwan University (SKKU) Suwon Republic of Korea; ^2^ Laboratoire De Physique et Chimie des Nano‐Objets UMR 5215 INSA CNRS Université de Toulouse Toulouse France; ^3^ Department of Physics and Chemistry Daegu Gyeongbuk Institute of Science and Technology (DGIST) Daegu Republic of Korea; ^4^ Sungkyunkwan Institute of Energy Science and Technology (SIEST) Suwon Republic of Korea; ^5^ Department of Display Engineering Sungkyunkwan University (SKKU) Suwon Republic of Korea; ^6^ Department of Future Energy Engineering Sungkyunkwan University (SKKU) Suwon Republic of Korea

**Keywords:** cadmium phosphide colloidal quantum dots, charge‐selective layer, infrared photodiodes, molecular oxygen adsorption, polarity switching

## Abstract

Colloidal quantum dots (CQDs) based on II–V semiconductors offer attractive optical absorption and carrier transport properties for infrared optoelectronics, yet their device‐relevant electronic behavior remains poorly understood. In particular, Cd_3_P_2_ CQDs have been constrained by limited control over nanocrystal growth and carrier polarity. Here, a materials‐to‐device study establishes polarity control in Cd_3_P_2_ CQD solids for infrared photodiodes. Precise regulation of oleic acid (OA) concentration during synthesis yields monodisperse Cd_3_P_2_ CQDs with suppressed nanocrystal fusion and photoluminescence quantum yields up to 62 %. Electrical measurements reveal an oxygen‐induced transition from *n*‐type to *p*‐type transport in Cd_3_P_2_ CQD films. Spectroscopic analysis and first‐principles calculations indicate that adsorbed oxygen generates surface acceptor states that drive Fermi‐level realignment. Building on these functional Cd_3_P_2_ CQD solids, a Cd_3_P_2_‐based homojunction CQD photodiode is demonstrated, in which Cd_3_P_2_ functions as both the infrared absorber and a charge‐selective layer. The resulting devices exhibit stable ambient operation, achieving a short‐circuit current density of 18 mA cm^−2^, an external quantum efficiency (EQE) of 24 %, and a fast temporal response of 23 ns under zero bias. These results identify surface‐driven polarity control as a viable design strategy for II–V CQD optoelectronics and position Cd_3_P_2_ CQDs as a promising platform for low‐power infrared conversion technologies.

## Graphical abstract

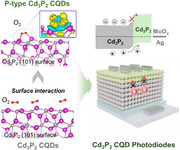

## 1 Introduction

Colloidal quantum dots (CQDs) have become a promising material for imaging, sensing, and energy‐conversion technologies owing to their solution processability, spectral tunability, and large‐area fabrication [1, 2, 3, 4, 5]. Beyond the extensively studied IV–VI and III–V CQD systems, II–V compound semiconductors represent an emerging and largely unexplored class of CQDs with distinctive promise for optoelectronics. Owing to their mixed covalent–ionic bonding and relatively light effective masses, II–V materials exhibit strong optical absorption and pronounced quantum confinement with the potential for high carrier mobility and narrow bandgaps—attributes that are particularly attractive for infrared optoelectronic applications [6, 7, 8].

Among II–V semiconductors, cadmium phosphide (Cd_3_P_2_) stands out as a promising candidate for near‐infrared optoelectronics. Bulk Cd_3_P_2_ exhibits a narrow bandgap (0.55 eV at 300K), moderately high dielectric constant (5.8) [9], strong near‐infrared absorption [10, 11], and *n*‐type bulk transport with relatively high carrier mobilities [12, 13, 14]. When reduced to the quantum‐confined regime, Cd_3_P_2_ CQDs additionally offer wide size‐tunable optical response and solution‐processable film formation, positioning them as a promising alternative to established infrared CQD systems. Despite these favorable intrinsic properties, Cd_3_P_2_ CQDs remain far less developed than their IV–VI and III–V counterparts. Fundamental aspects—including control over nanocrystal growth and fusion during synthesis, charge transport mechanisms in CQD solids, and the origin and tunability of carrier polarity—are still poorly understood. As a result, device‐level demonstrations of Cd_3_P_2_‐based CQD optoelectronics remain limited.

Polarity control in CQD assemblies is a central strategy for regulating charge transport and advancing device performance, yet it remains a particularly unresolved issue in Cd_3_P_2_ CQD solids. Previous studies on IV–VI and III–V CQD systems have shown that stoichiometry [15, 16, 17], surface chemistry [18, 19], and dopants [20, 21, 22] can be used to engineer band alignment and carrier type. In PbS QDs, cation‐rich surfaces and halide ligands favor *n*‐type transport [23], while ligand exchange with excess sulfide induces strong *p*‐type conduction [16]. Yoon et al. realized stable *p*‐type transport in InAs QDs by precise Zn‐mediated doping while maintaining a constant In:As stoichiometry [21]. In contrast, although bulk Cd_3_P_2_ is commonly *n*‐type due to phosphorus deficiency [12, 13, 14], CQD solids are dominated by surface‐to‐volume ratios orders of magnitude larger than in bulk crystals. In such systems, surface coordination, ligand chemistry, and ambient adsorption can dominate intrinsic defect chemistry in determining electronic behavior. However, whether—and how—carrier polarity in Cd_3_P_2_ CQDs can be modulated by surface interactions has not been systematically investigated.

First, we explored the synthetic parameters to obtain mono‐dispersed Cd_3_P_2_ CQDs for photodiode fabrication. By varying the oleic acid (OA) concentration during synthesis, we uncover a ligand‐deficient growth regime in which Cd_3_P_2_ CQDs are prone to fusion, likely due to incomplete surface passivation and the presence of stable intermediates. We establish a synthetic condition that suppresses fusion and produces monodisperse Cd_3_P_2_ CQDs, enabling reliable probing of intrinsic electronic behavior. We further reveal a reversible polarity switching from *n*‐type to *p*‐type transport induced by molecular oxygen adsorption in the CQD film. Through comprehensive spectroscopic analysis and theoretical considerations, we demonstrate that this polarity switching originates from oxygen‐induced surface interaction, which drives Fermi‐level realignment without any extrinsic dopants. Finally, we leverage this *p*‐type state to demonstrate a Cd_3_P_2_‐based CQD photodiode, in which Cd_3_P_2_ functions as an infrared absorber and a charge‐selective layer. This homojunction architecture enables enhanced charge extraction, high photocurrent density, and stable device operation. The resulting Cd_3_P_2_ CQD homojunctions exhibit a short‐circuit current density of 18 mA cm^−2^, an external quantum efficiency (EQE) of 24 %, and a fast response rate of 23 ns even without bias. By integrating controlled CQD synthesis, reversible polarity engineering, and device‐level integration, this work defines a pathway for polarity control in II–V CQD optoelectronics and highlights their potential for low‐power infrared energy‐conversion applications.

## 2 Results and Discussion

### 2.1 Synthesis and Fabrication of Conductive Cd_3_P_2_ CQD Films

**FIGURE 1:**
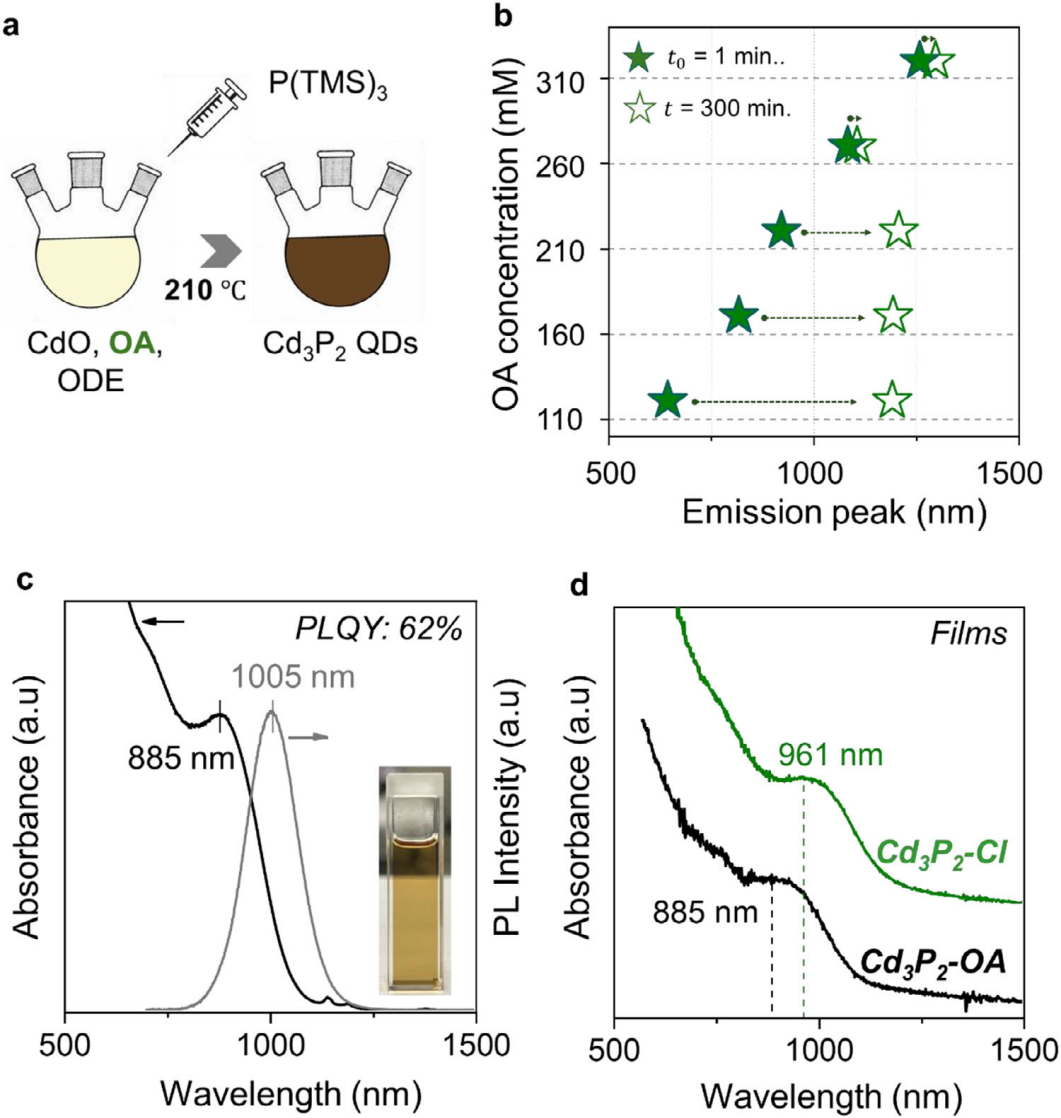
Synthesis and optical characteristics of Cd_3_P_2_ CQDs. (a) Schematic of the hot‐injection synthesis of Cd_3_P_2_ CQDs using CdO/OA in octadecene, with tris(trimethylsilyl)phosphine injected at 210°C. (b) Evolution of the photoluminescence (PL) peak position as a function of OA concentration, demonstrating the control over growth time. (c) UV–vis–NIR absorption (black) and PL (gray) spectra of Cd_3_P_2_ CQDs with first excitonic features centered at 885 and 1005 nm; inset shows a photograph of the colloidal dispersion. (d) Absorption spectra of Cd_3_P_2_ CQDs films before and after ligand exchange from oleate (Cd_3_P_2_‐OA) to chloride (Cd_3_P_2_‐Cl).

Achieving high monodisperses and colloidal stability is essential for reliably probing intrinsic charge transport in CQD solids and for realizing efficient CQD optoelectronic devices. First, we synthesize high‐quality cadmium phosphide (Cd_3_P_2_) CQDs via a hot‐injection route using in situ‐formed cadmium oleate (from CdO and OA) and tris(trimethylsilyl)phosphine (P(TMS)_3_) as the cadmium and phosphorus precursors [10, 11, 24], respectively (Figure 1a). We find that Cd_3_P_2_ CQD growth is highly sensitive to OA concentration, which modulates (i) the solubility of cadmium–carboxylate complexes and (ii) the extent of surface passivation that governs whether growth proceeds predominantly via monomer addition or via non‐classical pathways such as interparticle fusion/coalescence. Increasing OA produces a systematic redshift of the photoluminescence (PL) peak at early reaction times (Figure 1b), consistent with an increased solubility of monomers that lowers the nucleation concentration and favors the growth of larger nanocrystals [25, 26, 27, 28]. The enhanced ligand coordination further stabilizes growing surfaces, promoting sustained monomer addition rather than secondary nucleation [29, 30, 31, 32]. More importantly, OA concentration dictates the dominant growth pathway at extended reaction times: under ligand‐deficient conditions (OA< 260 mM), prolonged reactions lead to pronounced nanocrystal fusion/coalescence (Figure 1b), whereas at higher OA concentrations, fusion is effectively suppressed, yielding stable and monodisperse CQDs (Figure 1b).

Optical spectroscopy reveals a transient CQD population under low‐OA conditions (Figure S1a,b), evidenced by an emission band centered at 850 nm that coexists with a more redshifted population, resulting in a multimodal PL distribution. This 850 nm population is observed over a limited duration before gradually disappearing as interparticle fusion/coalescence becomes dominant [29, 33, 34, 35, 36]. Correspondingly, in the absorption spectra, the emergence of new absorption features coincides with the appearance of the second emission peak, indicating that the multimodal PL originates from fused CQDs formed during non‐classical growth, rather than from trap‐assisted radiative recombination. Moreover, transmission electron microscopy (TEM) images (Figure S2) show morphological evidence of nanocrystal–nanocrystal fusion at prolonged reaction times. When isolated, the 850 nm CQDs exhibit discrete optical features, narrow PL linewidths, high monodispersity, and air stability (Figure S3), supporting their identification as a stable intermediate rather than aggregates or scattering artifacts [37, 38, 39]. These results suggest that low OA concentrations promote the formation of stable nanocrystal intermediates, which in turn facilitate fusion‐mediated growth. In contrast, higher OA concentrations provide more effective surface coordination, stabilizing individual nanocrystals and enforcing a growth pathway dominated by monomer addition [35, 38, 40]. The full time‐resolved growth spectra of Cd_3_P_2_ CQDs synthesized with OA concentrations below and above 260 mM are shown in Figure S4. Under OA‐deficient conditions (160 and 210 mM), the presence of stable intermediate dots promotes dot‐to‐dot fusion, leading to the emergence of multimodal emission peaks. In contrast, at 260 mM OA, a single emission mode is observed and remains stable over time. These observations further support a non‐classical growth mechanism for Cd_3_P_2_ CQDs under OA‐deficient conditions.

Based on these insights, Cd_3_P_2_ CQDs synthesized at an OA concentration of 260 mM exhibit suppressed fusion and yield a monodisperse population with an emission peak at 1005 nm and a high photoluminescence quantum yield (PLQY) of 62 %, which are used for subsequent characterization and device studies. The corresponding absorption and photoluminescence spectra are shown in Figure 1c, with TEM images and XRD analysis provided in Figure S5.

Next, to investigate charge transport in Cd_3_P_2_ CQD films, the native long‐chain oleate ligands were replaced with halide ligands via solid‐state ligand exchange using tetrabutylammonium halides (TBAX, X═Cl, Br, I). In this process, insulating Cd–carboxylate surface coordination is disrupted and replaced by compact Cd–halide bonds, thereby reducing interparticle spacing and enhancing electronic coupling [41, 42, 43]. Fourier‐transform infrared spectroscopy (FTIR) analysis verifies the successful replacement of native oleate ligands with halide ions by the disappearance of ─CH_2_/─CH_3_ stretches at 2850 and 2920 cm^−1^ and the C─O stretching at 1510 cm^−1^ (Figure S6a). Among the halides, chloride produced the strongest redshift in absorption and the highest carrier mobility (Figure S6b,c), consistent with tighter inter‐dot packing enabled by the smaller ionic radius of Cl^−^ [43]. Film absorption of Cd_3_P_2_ CQD before and after ligand exchange using chloride ligand is shown in Figure 1d. Based on these results, chloride‐capped Cd_3_P_2_ CQDs were used for all subsequent transport and device studies.

### 2.2 *n‐p* Polarity Switching in Cd_3_P_2_ CQD Films

**FIGURE 2:**
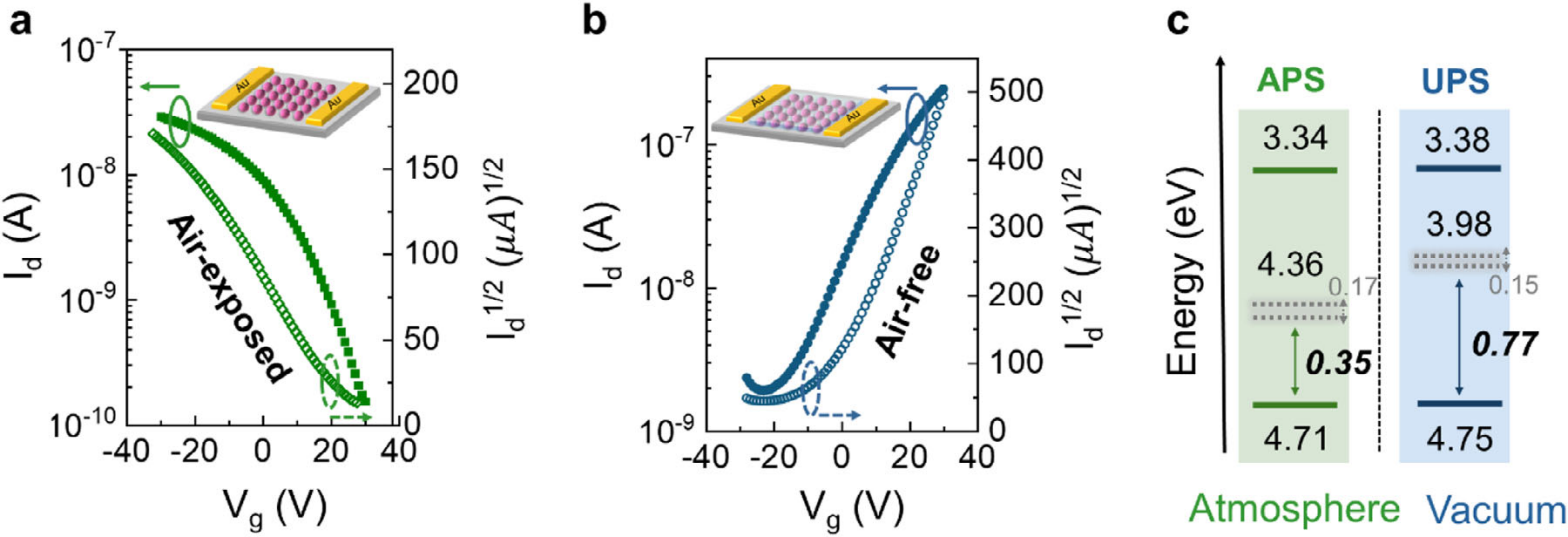
Polarity of Cd_3_P_2_ CQDs films. Transfer characteristics of Cd_3_P_2_ CQD FETs under (a) air‐exposed and (b) air‐free devices. (c) Comparison of UPS and APS measurements. The result reveals a downward shift of the Fermi level (dashed gray line) toward the VBM under ambient conditions.

Bottom‐gate Cd_3_P_2−_Cl CQD field‐effect transistors (FETs) were fabricated on Si/SiO_2_ substrates and measured under controlled environments. Unencapsulated devices displayed *p*‐type transfer characteristics (Figure 2a), whereas those encapsulated with poly (methyl methacrylate) (PMMA) exhibited *n*‐type conduction (Figure 2b). This finding is in strong contrast to the intrinsic *n*‐type polarity of bulk Cd_3_P_2_, which is well known to originate from phosphorus deficiency [13, 14]. Extracted FET parameters are summarized in Table 1. The full output/transfer curves and gate‐leakage characteristics are shown in Figure S7. Kelvin‐probe (KP) and ambient photoemission spectroscopy (APS) measurements corroborated this change, showing the Fermi level shifting toward the valence‐band maximum (VBM) upon air exposure. As presented in Figure 2c, under high vacuum, ultraviolet photoelectron spectroscopy (UPS) placed the Fermi level near the conduction band; under ambient conditions, APS revealed a ∼0.42 eV downward shift, indicating substantial Fermi‐level realignment driven by surface adsorption. The full UPS and KP/APS spectra are provided in Figure S8. In addition, to examine changes in surface chemistry upon air exposure, we performed XPS measurements on Cd_3_P_2_ CQD films in the as‐prepared state and after 5 min of air exposure, as shown in Figure S9. After air exposure, no noticeable changes are observed in the Cd 3d and P 2p spectra, indicating that the Cd chemical environment remains unchanged. This observation is consistent with the switchable polarity behavior observed in Cd_3_P_2_ CQD solids and suggests that it is not associated with irreversible chemical modification of Cd sites. We note that molecularly adsorbed oxygen can desorb under the high‐vacuum conditions of XPS measurements, which is consistent with the recovery of *n*‐type behavior observed in UPS.

**TABLE 1:** Extracted FET data of encapsulated and unencapsulated devices.

FET Cd_3_P_2_ CQDs	Carrier type	µ_*lin*_ (cm^2^V^−1^ s^−1^)	I_on/off_	V_T_ (V)	Carrier concentration (cm^−3^)
Encapsulated	n‐type	1.7 × 10^−5^	10^2^	7.2	2.1 × 10^17^
Unencapsulated	p‐type	1.0 × 10^−6^	10^2^	13.4	9.6 × 10^16^

To examine the reversibility of the polarity inversion, we performed controlled atmosphere switching experiments on Cd_3_P_2_ CQD FETs (Figure S10a). Devices exposed to ambient air exhibit clear *p*‐type transport, whereas subsequent thermal annealing (100°C, 5 min) under nitrogen followed by encapsulation restores *n*‐type behavior (Figure S10b,c). The annealing step is critical, as it promotes desorption of surface‐adsorbed species [44, 45] and recovers electron transport with increased mobility and on/off ratio (Figure S11). Upon removal of the encapsulation and re‐exposure to air, the devices revert to *p*‐type conduction (Figure S10d), confirming the reversibility of the process. Similar *n*‐type behavior is also observed when using a UV‐curable resin instead of PMMA as the encapsulant (Figure S12), indicating that suppression of ambient exposure–rather than the encapsulation material itself–governs carrier polarity. These results demonstrate that polarity switching in Cd_3_P_2_ CQD films is reversible and controlled by adsorption and desorption of ambient species at the CQD surface.

In addition, to evaluate whether the polarity inversion depends on nanocrystal dimension, we synthesized Cd_3_P_2_ QDs with mean diameters of 2.9, 4.1, and 5.5 nm. The QD sizes were confirmed by TEM images and shown in Figure S13. Their corresponding absorption and PL spectra are presented in Figure S14a. Each sample then underwent identical ligand exchange and FET fabrication. All sizes showed the same switching: *p*‐type under ambient exposure and *n*‐type when encapsulated (Figure S14b,c). Carrier mobilities were extracted and exhibited an increase monotonically with QD size. The enhanced mobility with size arises from reduced inter‐dot hopping barriers [46, 47, 48], while the identical polarity behavior confirms that the inversion mechanism is extrinsic rather than structural. As shown in Figure S15, X‐ray photoelectron spectroscopy (XPS) revealed slightly Cd‐rich surfaces for all samples, indicating that ambient molecule adsorption–not intrinsic stoichiometry–governs the *p*‐type transition.

**FIGURE 3:**
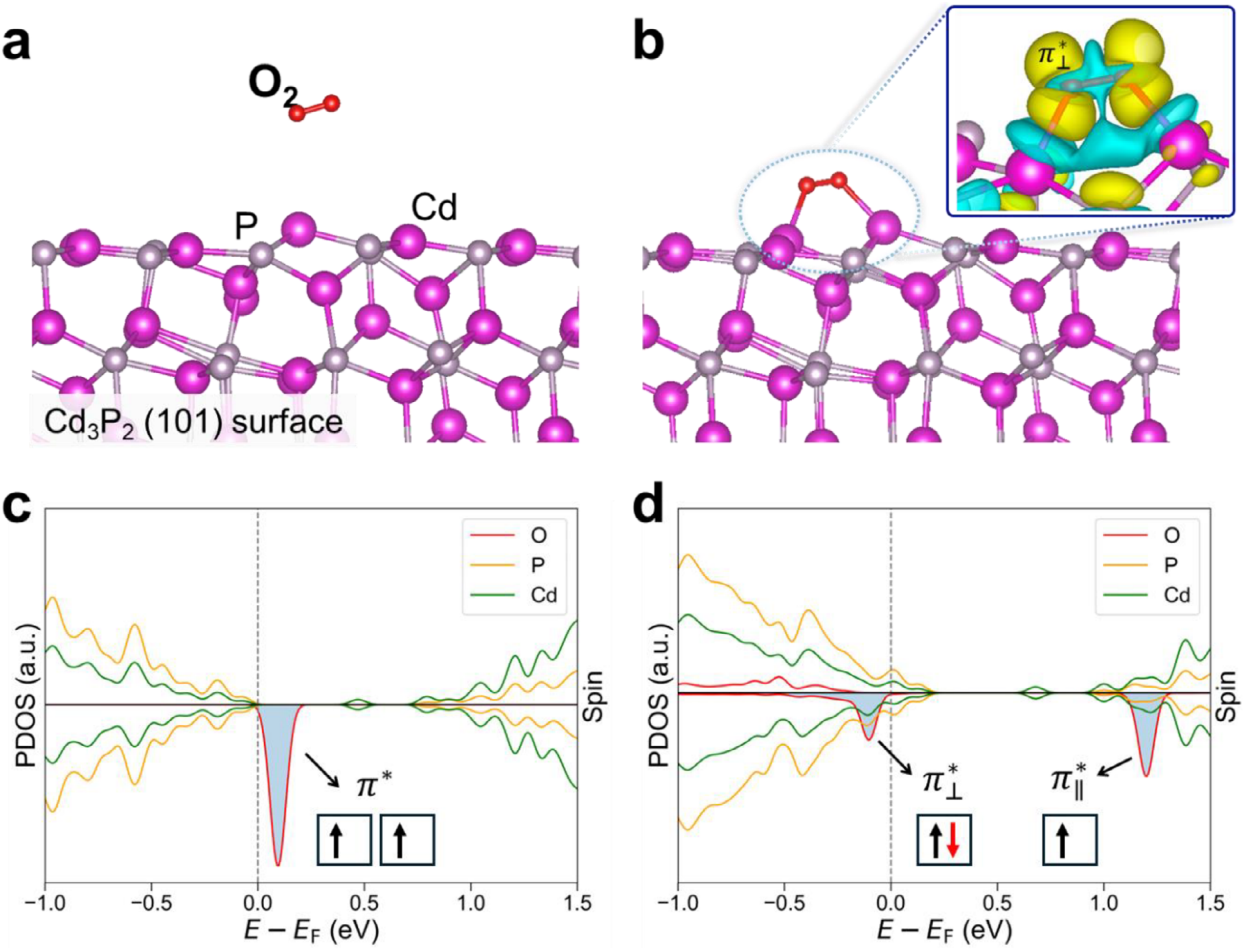
Mechanistic role of molecular oxygen adsorption on Cd_3_P_2_ surfaces. (a, b) DFT‐optimized structures of the Cd_3_P_2_ (101) surfaces (a) before and (b) after O_2_ adsorption. Inset in (b): charge density difference map showing electron transfer from the Cd_3_P_2_ surface into the $\pi_{⊥}^{*}$ antibonding orbital of the adsorbed O_2_ (yellow: electron accumulation; cyan: depletion). (c,d) Spin‐polarized partial density of states (PDOS) corresponding to (a,b). The Fermi level (*E*_F_) is set to zero. (d) Upon adsorption, the O_2_ $\pi_{⊥}^{*}$ spin‐down state becomes occupied and lies below the valence band maximum (VBM), while the $\pi_{∥}^{*}$ state remains unoccupied above the conduction band minimum (CBM).

These observations of reversible polarity switching in Cd_3_P_2_ QD films raise a fundamental question: Which molecular species is responsible for this behavior? To address this, we performed first‐principles calculations using Cd_3_P_2_ surface slab models (Figure 3). As mentioned earlier, XRD analysis (Figure S5) confirmed that the synthesized Cd_3_P_2_ QDs adopt a tetragonal crystal structure; accordingly, slab models were constructed from the tetragonal bulk phase. In spherical QDs, both polar and non‐polar surfaces coexist within the CQDs [49]. Polar surfaces are stabilized by ligand passivation, which sterically shields them from molecular interactions. In contrast, non‐polar surfaces are intrinsically stabilized through self‐passivation, eliminating dangling bonds but leaving surface atoms chemically accessible to adsorption or oxidation because of the absence of ligand passivation. Among the considered surface terminations (Table S1), the Cd_3_P_2_ (101) surface is the most thermodynamically stable and non‐polar, exhibiting a well‐defined bandgap (Figure S16). This combination makes the (101) surface a representative model for studying adsorption processes. Our DFT calculations identified multiple favorable binding sites and configurations for O_2_ adsorption (Figure 3b; Figure S17), with adsorption energies ranging from −0.79 to −0.34 eV. Such moderate binding strength suggests that O_2_ can desorb during annealing, consistent with the observed reversibility of polarity switching.

To reveal how O_2_ adsorption induces *p*‐type doping, we compared the electronic structure of the Cd_3_P_2_ (101) slab before and after adsorption (Figure 3a,b). Calculations were performed using the spin‐polarized meta‐generalized gradient approximation (meta‐GGA). When an O_2_ molecule is far from the surface, its two‐degenerate spin‐down π* antibonding orbitals remain unoccupied and lie above the valence band maximum (VBM) of Cd_3_P_2_ (Figure 3c). Upon O_2_ adsorption, interaction with the surface Cd atoms breaks the rotational symmetry around the O─O bond, lifting the two‐fold degeneracy; the $\pi_{⊥}^{*}$ orbital (oriented perpendicular to the surface) lies lower in energy than the in‐plane $\pi_{∥}^{*}$ orbital. The spin‐down $\pi_{⊥}^{*}$ orbital becomes occupied by accepting an electron from the Cd_3_P_2_ surface (Figure 3b,d). The Fermi level is lowered below the VBM, leaving holes in the valence band. Thus, the adsorbed oxygen acts as a shallow acceptor, enabling modulation of the energy‐level alignment and driving the *p*‐type polarity. This theoretical insight is also reflected in our UPS and KP/APS measurements.

We also considered adsorption of H_2_O, another abundant ambient species. On the Cd_3_P_2_ (101) surface, H_2_O forms a dative bond with a Cd surface atom, with an adsorption energy of −0.71 eV, comparable to that of O_2_. However, because H_2_O possesses a large HOMO–LUMO gap encompassing the Cd_3_P_2_ bandgap (Figure S18), it lacks electronic states near the Cd_3_P_2_ band edges. Consequently, H_2_O adsorption cannot induce polarity switching.

We experimentally assessed whether humidity contributes to the polarity switching by controlling the exposure atmosphere between a dry‐room environment (≈0 % RH, ambient O_2_) and laboratory ambient air (≈30 % RH, ambient O_2_). FETs were fabricated under inert conditions and then exposed within 5 min to each environment for controlled exposure, followed by thermal annealing (100°C, 5 min) and PMMA encapsulation before electrical characterization. In both environments, the devices exhibited the same *n*‐type dominant conduction after annealing/encapsulation (Figure S19)–indicating that within the tested range, water is not the primary driver of the polarity inversion. These results support the conclusion that switching is dominated by oxygen‐related surface interactions rather than humidity effects.

The consistent *p/n*‐type inversion across different sizes and encapsulation schemes reveals the surface‐driven nature of transport in II–V CQD assemblies. The identified oxygen‐adsorption mechanism had not previously been verified in Cd_3_P_2_ nanocrystals. Because the process is both mild and reversible, it enables post‐fabrication tuning of device polarity without chemical doping. This feature could be exploited for adaptive photodetectors or reconfigurable electronic interfaces responsive to ambient conditions.

### 2.3 Photodiode Fabrication Based on Cd_3_P_2_ CQDs

**FIGURE 4:**
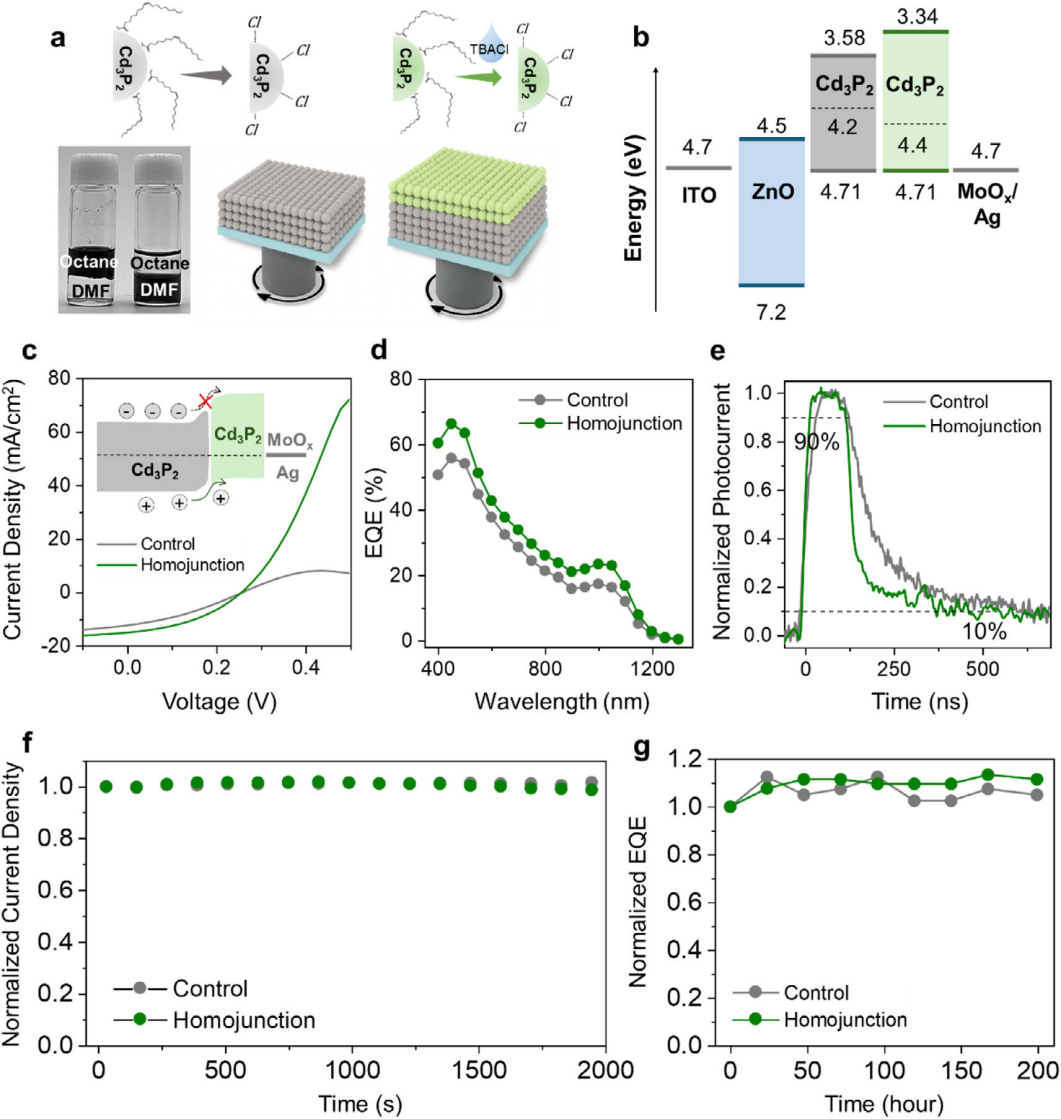
Performance of Cd_3_P_2_ CQD photodiodes. (a) Schematic illustration of ink processing and device architecture, highlighting the introduction of a *p*‐type Cd_3_P_2_‐Cl interlayer between the Cd_3_P_2_ absorber and the MoO_x_/Ag anode; photographs of phase separation before and after ligand exchange. (b) Energy‐level alignment of the device stack. (c) Current density–voltage (*J–V*) characteristics under illumination, (d) EQE spectra and (e) transient photoresponse of control devices and homojunction devices. (f) Operational stability under maximum power point (MPP) tracking, and (g) ambient stability assessed by EQE at −0.5V of unencapsulated devices.

Using the successful development of high‐quality, monodisperse Cd_3_P_2_ CQDs and their functional solid‐state polarity properties, we designed an infrared photodiode architecture composed of Cd_3_P_2_ CQD materials. For the main light‐absorbing layer, we developed a solution‐phase ligand exchange process for Cd_3_P_2_ CQDs, enabling a single‐step drop‐casting method to form a thick absorber layer of about 100 nm. By combining NOBF_4_ ligand stripping with ZnCl_2_ surface passivation, a clear phase transfer from octane to DMF is achieved, confirming efficient ligand exchange while preserving the peak‐to‐valley features of the pristine CQDs (Figure 4a; Figure S20). We then constructed the homojunction by introducing an additional *p*‐type Cd_3_P_2_ CQD solid, which supports the light absorption and facilitates hole extraction, aiming to realize charge selectivity through an internal junction formed at the interface.

The energy‐level alignment (Figure 4b) reveals a favorable band offset between the Cd_3_P_2_ main active layer and the *p*‐type Cd_3_P_2_‐Cl solid, giving rise to an internal junction within the same material system. This homojunction configuration is expected to provide efficient light absorption while enabling selective charge extraction. By comparing control devices without the junction (ZnO/Cd_3_P_2_ absorber/MoO_x_/Ag) and with the homojunction devices (ZnO/Cd_3_P_2_ absorber/*p*‐Cd_3_P_2_‐Cl/MoO_x_/Ag), we evaluate whether the *p*‐type Cd_3_P_2_ CQD solid can facilitate the hole extraction in the photodiodes.

We first measure the current–voltage (*J*–*V*) characteristics under illumination to evaluate the photodiode behavior (Figure 4c). Interestingly, the control photodiode already exhibits a high short‐circuit current density (J_sc_) of 12.6 mA cm^−2^, which is a comparable value typically reported for III–V CQD photodiodes and underscores the strong optical absorption and efficient carrier generation in Cd_3_P_2_ CQDs (Table S2). Notably, upon insertion of the *p*‐type Cd_3_P_2_‐Cl layer, the rectification ratio is significantly improved, which can be attributed to enhanced charge selectivity at the diode interface [50]. Correspondingly, the J_sc_ increases substantially to 18.0 mA cm^−2^, accompanied by improvements in both the fill factor and the open‐circuit voltage. The extracted photovoltaic parameters are summarized in Table 2.

**TABLE 2:** Photovoltaic performance parameters of Cd_3_P_2_ CQD photodiodes, control, and homojunction devices.

Device	J_sc_ (mA/cm^2^)	V_oc_ (V)	FF (%)	PCE (%)
Control	12.5 ± 0.08	0.21 ± 0.01	36.75 ± 1.23	0.97 ± 0.04
Homojunction	17.98 ± 0.04	0.23 ± 0.01	39.25 ± 0.48	1.63 ± 0.05

Consistent with this interpretation, the EQE spectra (Figure 4d) exhibit an overall enhancement across the entire spectral range at zero bias. In particular, the short‐wavelength EQE increases markedly from ∼55 % to nearly 70 %, while the EQE at the first excitonic peak improves from 17 % to 24 % in the homojunction structure. These enhancements indicate a substantial increase in the internal drift current, reflecting more efficient charge separation and collection enabled by improved interfacial selectivity [18, 19, 50]. The achievement of a 24 % EQE and J_sc_ of 18.0 mA cm^−2^ at zero applied bias represents a notably high value compared to previously reported infrared photodiodes based on III–V or silver chalcogenides (e.g., Ag_2_Te), highlighting the potential of II–V material–based devices (Table S2)

Importantly, the improved interfacial energetics also translate into significantly faster photoresponse dynamics. As shown in Figure 4e, a markedly fast response is achieved even under zero applied bias upon insertion of the *p*‐type Cd_3_P_2_ layer, with the rise time decreasing from ∼40 to ∼23 ns and the fall time from ∼475 to ∼250 ns compared to the control device. These results highlight the role of polarity‐engineered charge selectivity in accelerating carrier extraction and minimizing carrier accumulation at the electrode interface. Based on the fast photoresponse, we further evaluated the device performance as a photodetector (Figure S21). Owing to the efficient charge extraction, the device achieves complete carrier collection at relatively low operating bias. As a result, a high specific detectivity (D^*^) of approximately 1.32 × 10^11^ Jones is obtained at −0.5 V, demonstrating high‐sensitivity detection under low‐power operation with low dark current.

The Cd_3_P_2_ CQD photodiodes further exhibit operational and air stability, retaining over 98 % of their initial output during 2000 s of maximum power point tracking and 100 % of EQE after 200 h under ambient conditions without encapsulation (Figure 4f,g). This combination of high photocurrent density, efficient charge selectivity, fast temporal response, and high stability highlights the potential of II–V CQD based photodiode as a versatile platform for high‐performance, energy‐efficient optoelectronic applications.

## 3 Conclusions

In conclusion, this work establishes Cd_3_P_2_ QD solids as an environmentally driven semiconductor platform whose electronic polarity can be reversibly tuned through surface‐interaction engineering. By constructing a monodisperse Cd_3_P_2_ CQD system with well‐defined surface chemistry, we uncover a robust polarity inversion from *n*‐type to *p*‐type transport upon oxygen exposure. Spectroscopic analysis and theoretical modeling show that this transition originates from oxygen‐induced surface dipoles that realign the Fermi level. Leveraging this polarity‐engineered *p*‐type state, we demonstrate a dopant‐free, charge‐selective architecture in quantum dot photodiodes, in which band‐offset‐mediated charge extraction enables enhanced photocurrent density and stable operation under ambient conditions without encapsulation. The resulting devices achieve a short‐circuit current density of 18 mA cm^−2^, an EQE of 24 %, and a fast response dynamic of 23 ns under zero bias. Beyond the specific devices demonstrated here, this study establishes tunable electronic polarity in II–V CQDs, opening new opportunities for systematic investigation and device design in II–V CQD optoelectronics.

## 4 Experimental Section

### 4.1 Materials

Cadmium oxide (CdO, 99.99%), oleic acid (OA, 99%), tetrabutylammonium iodide (TBAI, 99.999%), tetrabutylammonium bromide (TBABr, 99.999%), tetrabutylammonium chloride (TBACl, 99.999%), nitrosonium tetrafluoroborate (NOBF_4_, 95%), zinc (II) chloride (ZnCl_2_, 97%), poly(methyl methacrylate) (PMMA, Mw: 15.000), methyl acetate (MeOAc, anhydrous, 99.5%), methanol (MeOH, anhydrous, 99%), toluene (anhydrous, 99.9%), octane (anhydrous, 99.9%) were purchased from Sigma Aldrich and used without any further modification. Tris(trimethylsilyl)phosphine (P(TMS)_3_, 95%) was purchased from SK Chemical, and 1‐octadecene (ODE, 99.999%) was supplied by Uniam. ODE was degassed under vacuum at 110°C for 10 h before use. All chemicals were stored in an inert environment.

### 4.2 Cadmium Phosphide QDs Synthesis

Cd_3_P_2_ CQDs were synthesized following a modified hot‐injection method based on previous reports [10]. In a typical procedure, 1.5 mmol of cadmium oxide (CdO), 25 mL of 1‐octadecene (ODE), and a specified amount of OA were loaded into a three‐neck flask and degassed under vacuum at 110 °C for 30 min. The OA amount was varied to control the CQD size from 3.2 to 9.5 mmol. The phosphorous precursor was prepared by dissolving 145 µL (TMS)_3_P in 2.5 mL ODE. Subsequently, phosphorous solution was injected into the cadmium precursor solution at 210°C under a nitrogen atmosphere. After a desired reaction time, the mixture was rapidly cooled to room temperature by removing the heating mantle. The synthesized Cd_3_P_2_ CQDs were purified using a mixed solvent of toluene and methyl acetate (MeOAc) in a 1:4 volume ratio. The purification process was performed at 11.000 rpm for 10 min and repeated three times prior to further use.

### 4.3 Solution‐State Ligand Exchange

Ligand exchange of Cd_3_P_2_ CQDs using nitrosonium tetrafluoroborate (NOBF_4_) was carried out following previously reported procedures [18]. Briefly, purified Cd_3_P_2_ CQDs were treated with NOBF_4_ to remove native oleate ligands, yielding ligand‐stripped (“naked”) Cd_3_P_2_ CQDs dispersed in dimethylformamide (DMF). Subsequently, a ZnCl_2_ solution (1 mM in DMF) was added to the naked Cd_3_P_2_ CQD solution, followed by stirring for less than 2 min to allow surface coordination. The CQDs were then precipitated by the addition of toluene, followed by centrifugation at 6000 rpm for 4 min. The resulting precipitate was dried under vacuum and subsequently redispersed in a mixed solvent of DMF and acetonitrile (ACN).

### 4.4 Solid‐State Ligand Exchange

The ligand exchange process was carried out in an inert environment. As‐synthesized Cd_3_P_2_ QDs (30 mg/mL in octane) were spin‐coated at 2500 rpm for 25 s, followed by the deposition of TBACl solution at a concentration of 10 mg/mL and soaking for 30 s. The film was then spin‐coated at 2500 rpm for 10 s and rinsed with MeOH (repeated three times). This exchange process was repeated twice to achieve a final thickness of 30 nm for FET devices. The films were then annealed at 150°C for 15 min.

### 4.5 FET Fabrication and Measurement

Bottom‐contact FETs were fabricated on heavily p‐doped silicon wafers (B‐doped Si) with a 300 nm SiO_2_ layer serving as the gate dielectric. The substrates featured pre‐patterned interdigital Au electrodes (channel width: 300 µm; channel length: 5 µm) acting as source and drain contacts. Cd_3_P_2_ QDs were deposited onto the substrate via spin coating, followed by solid‐state ligand exchange as described in the Ligand Exchange section. After deposition, the devices were briefly removed from the glovebox to gently clean residual QDs from the electrode regions using acetone under ambient conditions and subsequently transferred to a vacuum probe station (VPS) for electrical characterization under a base pressure of 4 mTorr. For encapsulated devices, poly (methyl methacrylate) (PMMA) was dissolved in toluene (10 mg/mL) and spin‐coated onto the QD films to form a protective overlayer, followed by annealing at 100°C for 5 min prior to removal from the glovebox.

### 4.6 Photodiode Fabrication

A sol–gel ZnO precursor solution was prepared by dissolving zinc acetate dihydrate (500 mg) and ethanolamine (143 µL) in 2‐methoxyethanol (5 mL), followed by stirring at room temperature for 12 h to ensure complete complexation. The ZnO electron transport layer (ETL) was deposited by spin coating the precursor solution onto the cleaned ITO substrates at 3000 rpm for 30 s and subsequently annealed at 200°C for 30 min in ambient air. This deposition–annealing cycle was repeated to obtain a bilayer ZnO ETL. Following ZnO deposition, the substrates were transferred into a nitrogen‐filled glovebox for CQD film processing. Cd_3_P_2_ CQD absorber layers were deposited by spin coating from ink solutions, followed by a brief thermal treatment at 150°C for 10 s. Subsequently, a ∼30 nm‐thick Cd_3_P_2_‐Cl film prepared via solid‐state ligand exchange was spin‐coated atop the absorber layer. Finally, a molybdenum oxide (MoO_x_, 10 nm) hole extraction layer and a silver (Ag, 120 nm) top electrode were sequentially deposited by thermal evaporation through a patterned shadow mask to complete the device fabrication. The devices were not encapsulated and were subsequently used for further characterization under ambient air.

### 4.7 Characterizations

The absorption spectra were monitored with a UV–vis–near‐infrared spectrophotometer (Shimadzu, UV3600). The photoluminescence spectra were collected from an absolute photoluminescence spectrometer (Hamamatsu, C9920‐02). The size and morphology of QDs were observed using a transmission electron microscope (JEOL, JEM ARM 200F). Surface chemistry composition, and energy level were characterized by X‐ray photoemission spectroscopy (XPS, ThermoFisher Scientific, NEXSA) and ultraviolet photoelectron spectroscopy (UPS, ThermoFisher Scientific, NEXSA). Kelvin probe (KP) and ambient photoemission spectroscopy (APS; APS04, KP Technology) were employed to assess the work function and valence band maximum of CQD films under air exposure. Crystal structure analysis was conducted using X‐ray diffraction (XRD; Rigaku SmartLab). Electrical measurements of FET devices were carried out in a vacuum probe station (VPS, VPX‐77H) under a pressure of 4 mTorr. Current density–voltage (*J*–*V*) characteristics of photodiodes were measured under simulated AM 1.5G illumination (100 mW cm^−2^, 1 sun) using a solar simulator equipped with a xenon arc lamp. EQE spectra were acquired using a monochromated light source coupled to a calibrated detector system. Transient photocurrent (TPC) measurements were carried out using a high‐speed digital oscilloscope with a bandwidth of 100 MHz to evaluate the temporal photoresponse of the devices (0.2 mm^2^ in area)

## 5 Theoretical Section

### 5.1 Computational Details

All first‐principles DFT calculations were performed using the projector‐augmented wave (PAW) method, as implemented in the Vienna ab initio simulation package [51, 52]. The Local‐density approximation (LDA) [53] was used for atomic structure optimization, and the r^2^SCAN meta‐generalized gradient approximation (meta‐GGA) functional [54] was employed for electronic structure calculations. A plane‐wave basis set with a kinetic energy cutoff of 400 eV was used. All atoms were fully relaxed until the residual forces were below 0.02 eV Å^−1^. For the *k*‐point sampling, we used 3 × 3 × 1 mesh for the Cd_3_P_2_ (001) surface, 3 × 2 × 1 mesh for the (100) surface, 2 × 2 × 1 mesh for the (110) surface, 2 × 3 × 1 mesh for the (101) surface, and 2 × 2 ×1 mesh for the (111) surface. For each surface, the slab thickness was increased until the convergence of the surface energy was achieved within 1 meV per cell. A vacuum region of approximately 15 Å was tested to be large enough to avoid any interactions between periodic slabs. The calculated lattice parameters for bulk tetragonal Cd_3_P_2_ were a = b = 8.559 Å, and c = 12.247 Å. The r^2^SCAN functional yields a bandgap of 0.47 eV. The calculated lattice parameters and bandgap show good agreement with the experimental values [13, 55, 56].

## Conflicts of Interest

The authors declare no conflicts of interest.

## Supporting information




**Supporting File**: advs74612‐sup‐0001‐SuppMat.docx.

## Data Availability

The data that support the findings of this study are available in the supplementary material of this article.
