# Supplementary material for: Surface‐Interaction‐Driven Polarity Switching in II–V Cd3P2 Colloidal Quantum Dots for Infrared Photodiodes
Source: Adv Sci (Weinh). 2026 Mar 2;13(27):e00061. doi: 10.1002/advs.202600061 (PMC13170213; doi:10.1002/advs.202600061)
Supplement: Supplementary file 1 — Supporting File: advs74612‐sup‐0001‐SuppMat.docx. [file ADVS-13-e00061-s001.docx]

**Supporting Information**

Surface-Interaction-Driven Polarity Switching in II-V Cd_2_P_3_ Colloidal Quantum Dots for Infrared Photodiodes

*Ha-Chi V. Tran^1✝^, Doeun Shim^1✝^, Youngsang Park^1✝^, Mahnmin Choi**^1^, Hyeonjun Jeong^1^, Guillaume Bonifas^2^, Liyan Ouyang^2^, Celine Nayral^2^, Fabien Delpech^2^, Joongoo Kang^3^, and Sohee Jeong^1,4,5,6*^*

^1^Department of Energy Science (DOES), Sungkyunkwan University (SKKU), Suwon 16419, Republic of Korea

^2^ Université de Toulouse, INSA, CNRS, Laboratoire de Physique et Chimie des Nano-Objets UMR 5215, 135 avenue de Rangueil F-31077 Toulouse cedex 4, France

^3^Department of Physics and Chemistry, Daegu Gyeongbuk Institute of Science and Technology (DGIST), Daegu 42988, Republic of Korea

^4^Sungkyunkwan Institute of Energy Science and Technology (SIEST), Suwon 16419, Republic of Korea

^5^Department of Display Engineering, Sungkyunkwan University (SKKU), Suwon 16419, Republic of Korea

^6^Department of Future Energy Engineering, Sungkyunkwan University (SKKU), Suwon 16419, Republic of Korea

^✝^These authors contributed equally.

^*^E-mail: s.jeong@skku.edu

**Figure S1. Time-resolved optical and morphological evolution of Cd_3_P_2_ CQDs synthesized under low oleic acid (OA) concentration (160 mM).** (a) Normalized absorption spectra collected at different reaction times (1–300 min), showing the evolution of excitonic features during extended growth under ligand-deficient conditions. (b) Corresponding photoluminescence (PL) spectra, revealing the transient appearance of a spectrally distinct emission band centered at 850 nm that coexists with a red-shifted population at intermediate reaction times, resulting in a multimodal PL distribution.


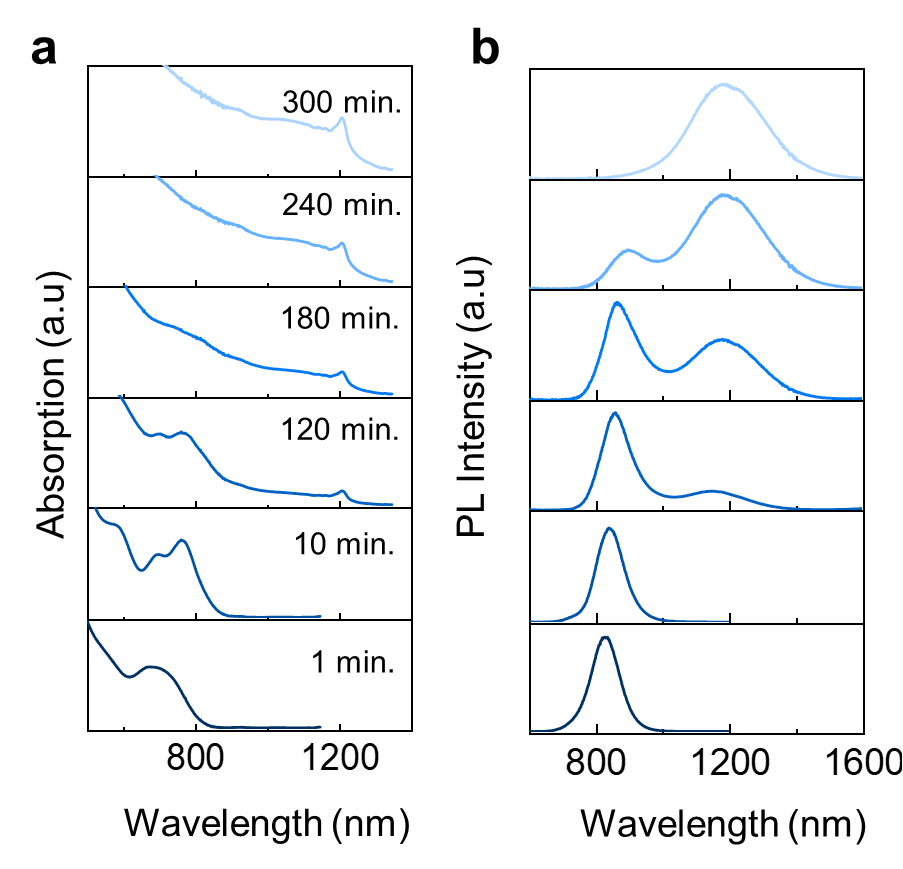

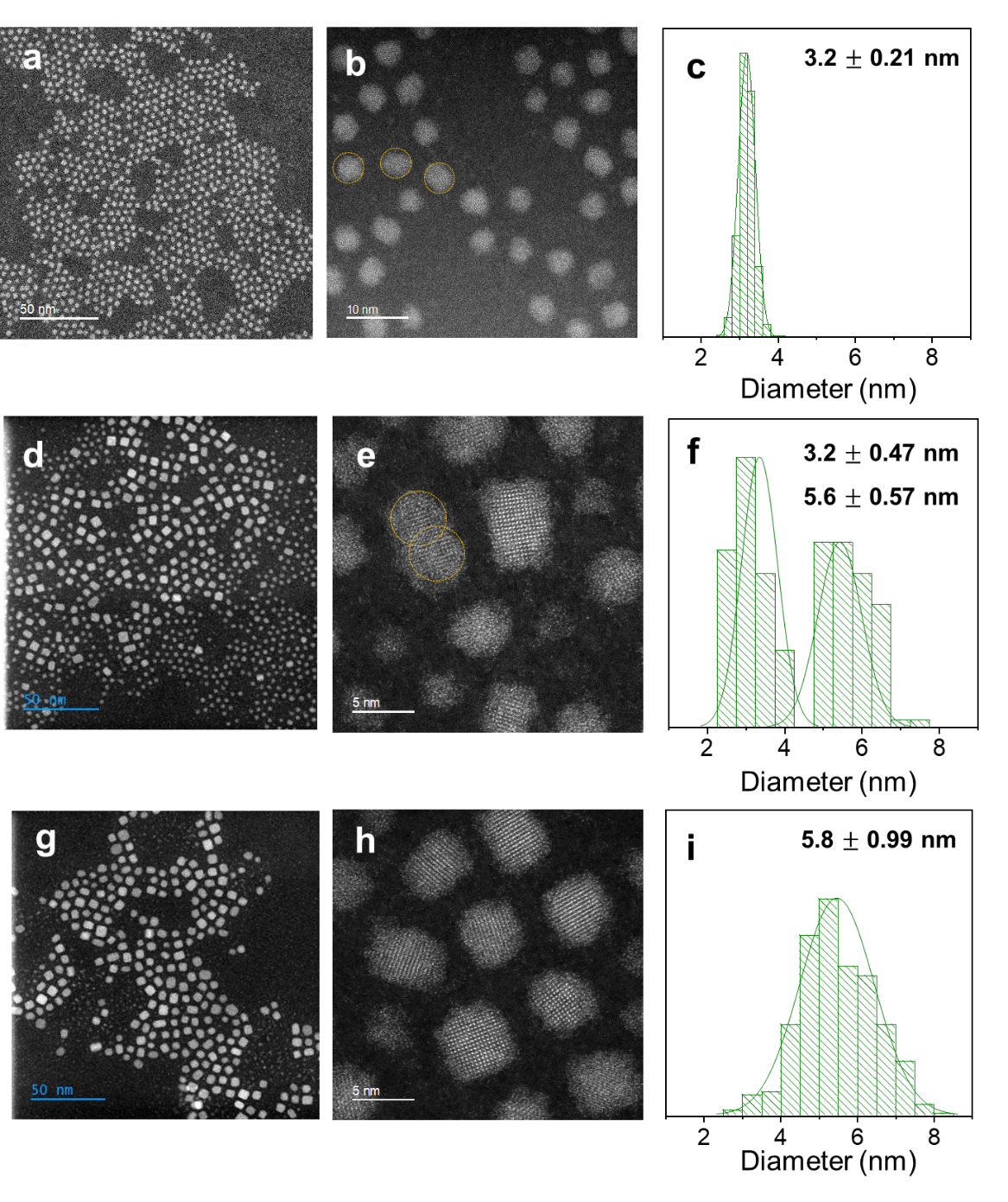


Figure S2. Transmission electron microscopy (TEM) analysis of Cd_3_P_2_ CQDs synthesized at 160 mM OA. (a–c) Low- and high-magnification TEM images and corresponding size distribution histogram of Cd_3_P_2_ CQDs synthesized at early reaction times (10 minutes). (d–f) TEM images and size histogram of Cd_3_P_2_ CQDs synthesized after 180 minutes, where pronounced nanocrystal fusion/coalescence is observed. (g–i) TEM images and size distribution of Cd_3_P_2_ QDs synthesized at 300 minutes.


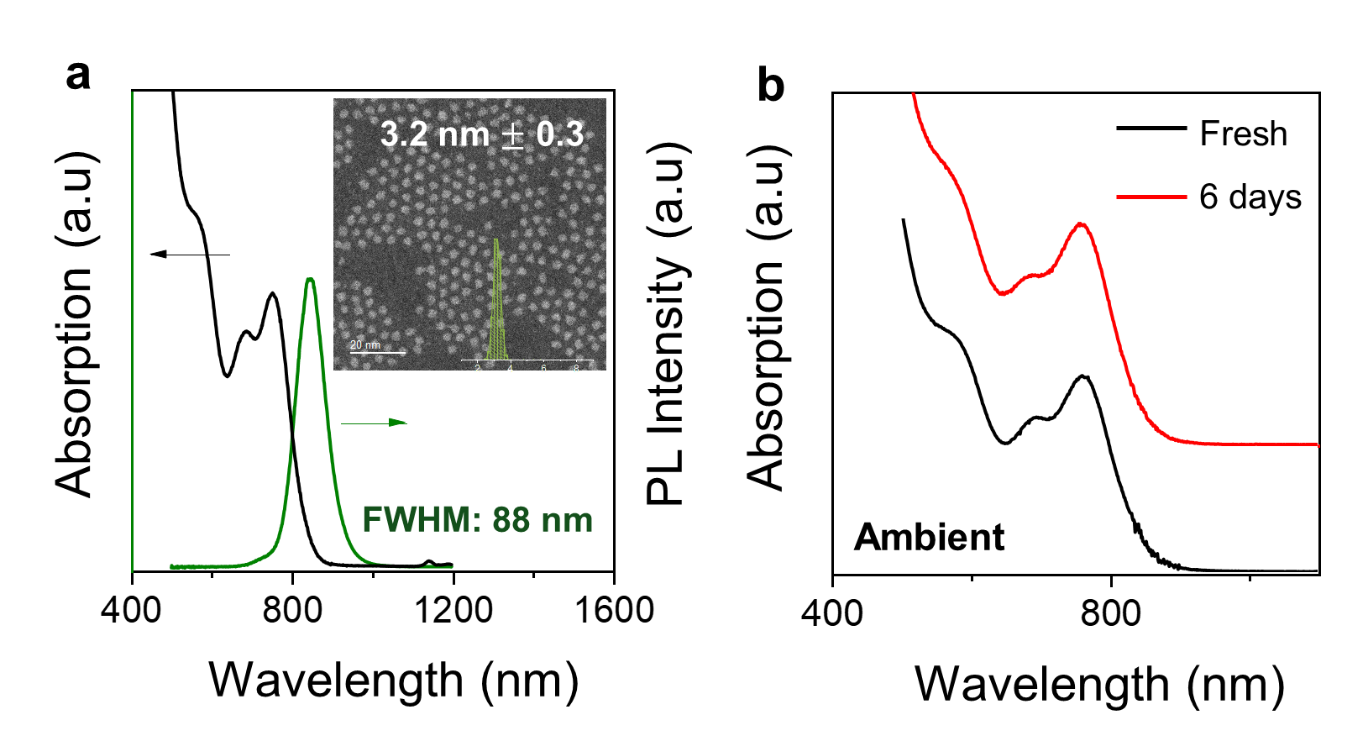


**Figure S3 Optical and structural characterization of the 850 nm Cd_3_P_2_ CQD population isolated from low-OA growth conditions.** (a) Absorption (black) and photoluminescence (green) spectra of the isolated 850 nm CQDs, exhibiting a well-defined excitonic transition and a narrow PL linewidth (FWHM ≈ 88 nm), indicative of high monodispersity; inset shows a TEM image with an average diameter of 3.2 ± 0.3 nm. (b) Absorption and PL spectra of the same CQDs measured immediately after purification (fresh) and after 6 days of storage under ambient conditions, demonstrating preserved spectral features and air stability.


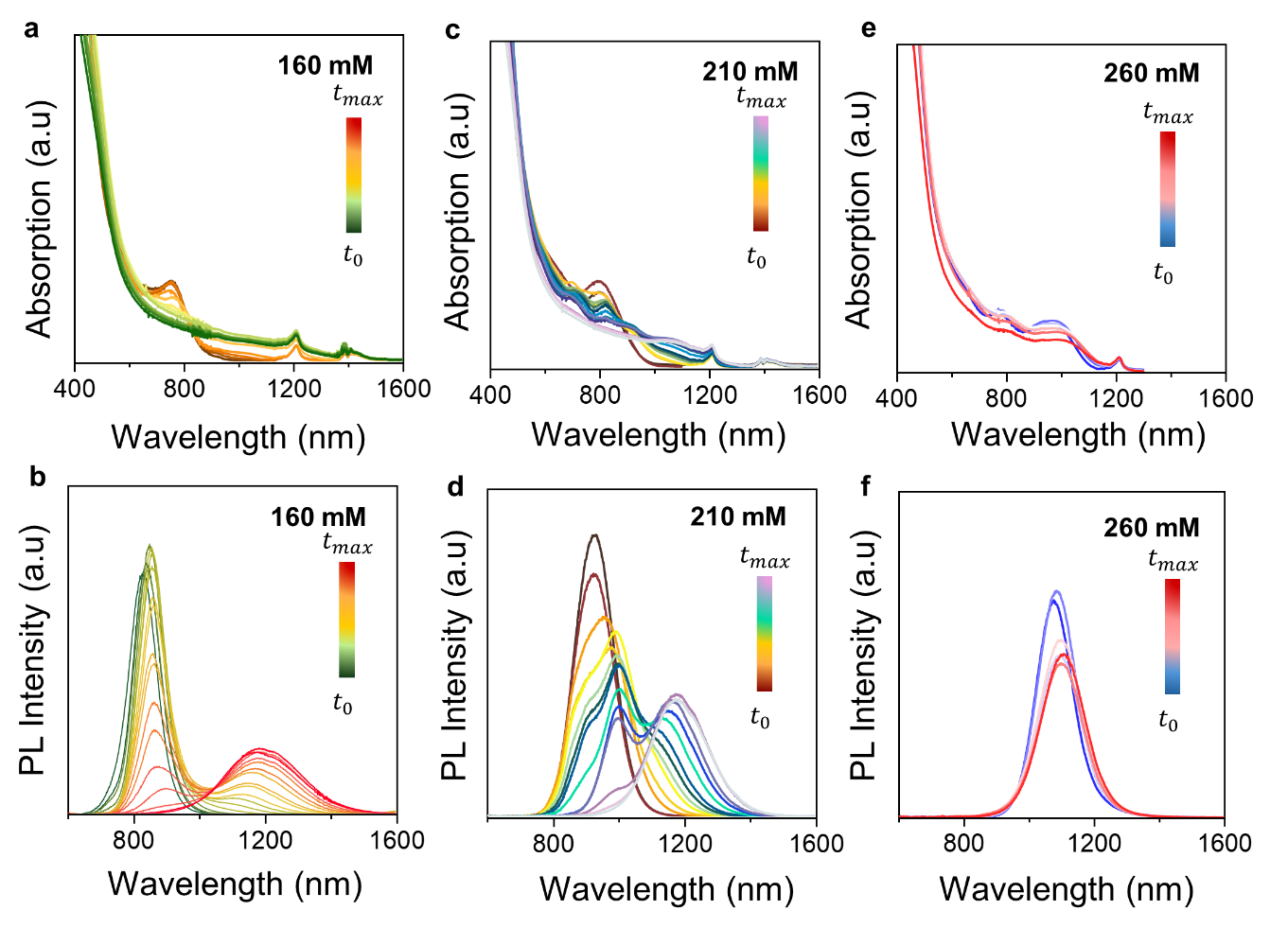


Figure S4. Time-dependent optical evolution of Cd_3_P_2_ CQDs synthesized under ligand-deficient and optimized oleic acid (OA) conditions (160 mM, 210 mM and 260 mM). (a, b) Absorption spectra (a) and corresponding photoluminescence (PL) spectra (b) of Cd_3_P_2_ CQDs synthesized under 160 mM-OA conditions, recorded at different reaction times (color-coded). (c, d) Absorption (c) and PL (d) spectra of Cd_3_P_2_ CQDs synthesized at 210 mM-OA concentration. (e, f) Absorption (e) and PL (f) spectra of Cd3P2 CQDs synthesized at 210 mM-OA concentration.


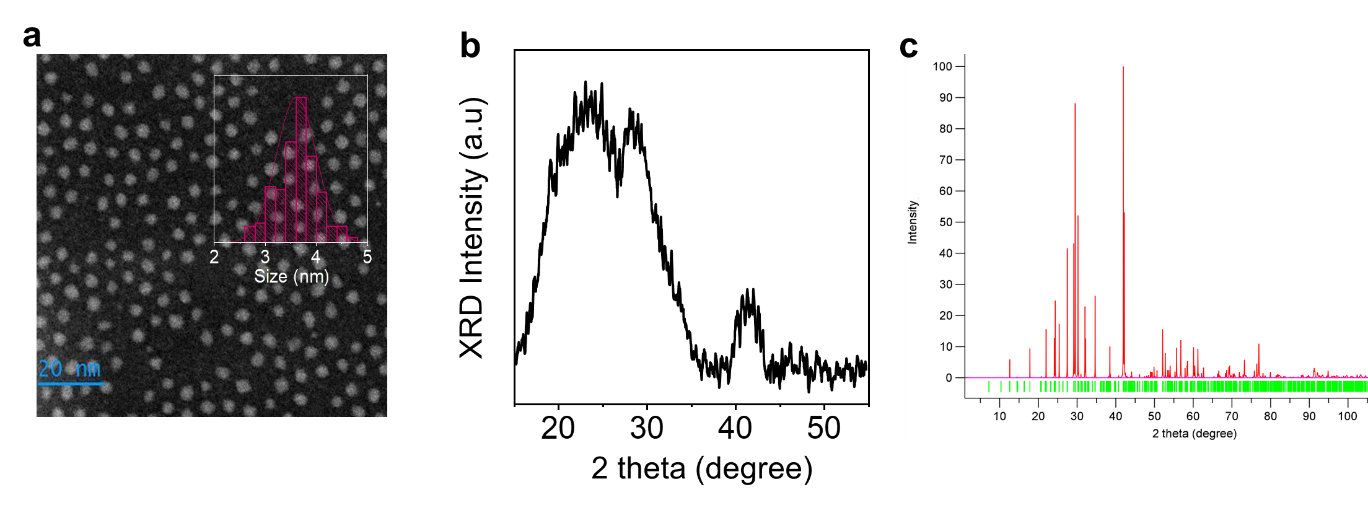


**Figure S5 Structural characterization of Cd_3_P_2_ CQDs synthesized at an oleic acid concentration of 260 mM.** (a) Transmission electron microscopy (TEM) image showing a uniform and well-dispersed Cd_3_P_2_ CQD ensemble; inset presents the corresponding size distribution histogram, indicating a narrow diameter distribution centered at 3.6 $\pm$ 0.5 nm. (b) X-ray diffraction (XRD) pattern of the Cd_3_P_2_ CQDs, exhibiting the tetragonal crystal structure. (c) Simulated XRD pattern of tetragonal Cd_3_P_2_. Powder XRD simulations were performed in VESTA using our DFT-calculated bulk structure. The simulated patterns were generated assuming Cu Kα radiation (λ = 1.5406 Å).


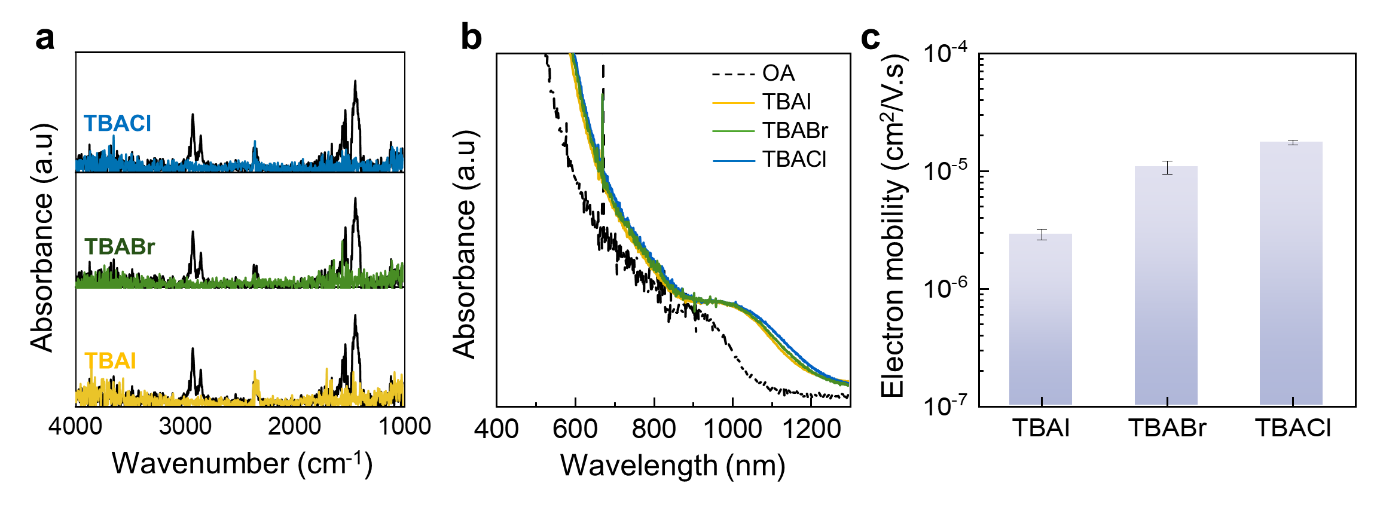


**Figure S6. Comparation of optical and electronic characterization of 885 nm-Cd_3_P_2_ QDs before and after ligand exchange with tetrabutylammonium halides (TBAX, X = I, Br, Cl).** (a) FTIR spectra confirming the successful replacement of native oleate ligands. (b) Absorption spectra of films after solid-state ligand exchange with TBAI, TBABr, and TBACl, compared to the native OA-capped QDs. (c) Extracted electron mobilities from FET measurements under Vds = 30V.


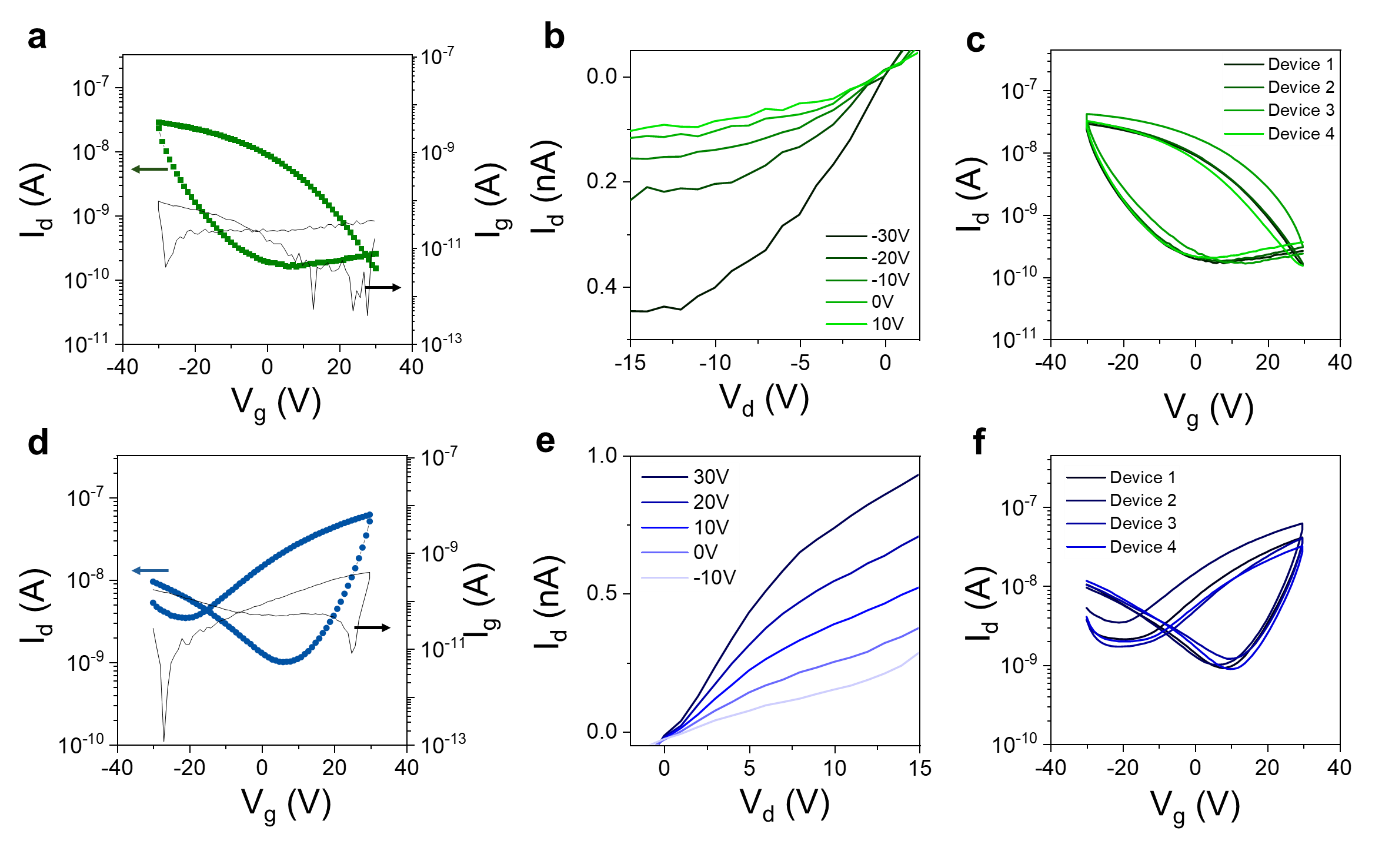


**Figure S7 Electrical characteristics of Cd_3_P_2_–Cl CQD field-effect transistors under different environmental conditions.** (a) Transfer characteristics (I_d_ vs V_g_) of an unencapsulated device, exhibiting dominant p-type transport; the gate leakage current (I_g_) is plotted on the secondary axis. (b) Corresponding output characteristics (I_d_ vs V_d_) measured at different gate voltages. (c) Transfer characteristics of multiple unencapsulated devices (Devices 1–4), demonstrating device-to-device reproducibility of the p-type behavior under ambient conditions. (d) Transfer characteristics of the same device after thermal annealing and encapsulation, revealing recovery of n-type transport behavior with suppressed gate leakage. (e) Output characteristics of the encapsulated device, confirming stable n-type conduction and systematic current modulation with gate bias. (f) Transfer characteristics of multiple encapsulated devices (Devices 1–4), showing reproducible n-type behavior following isolation from ambient exposure.


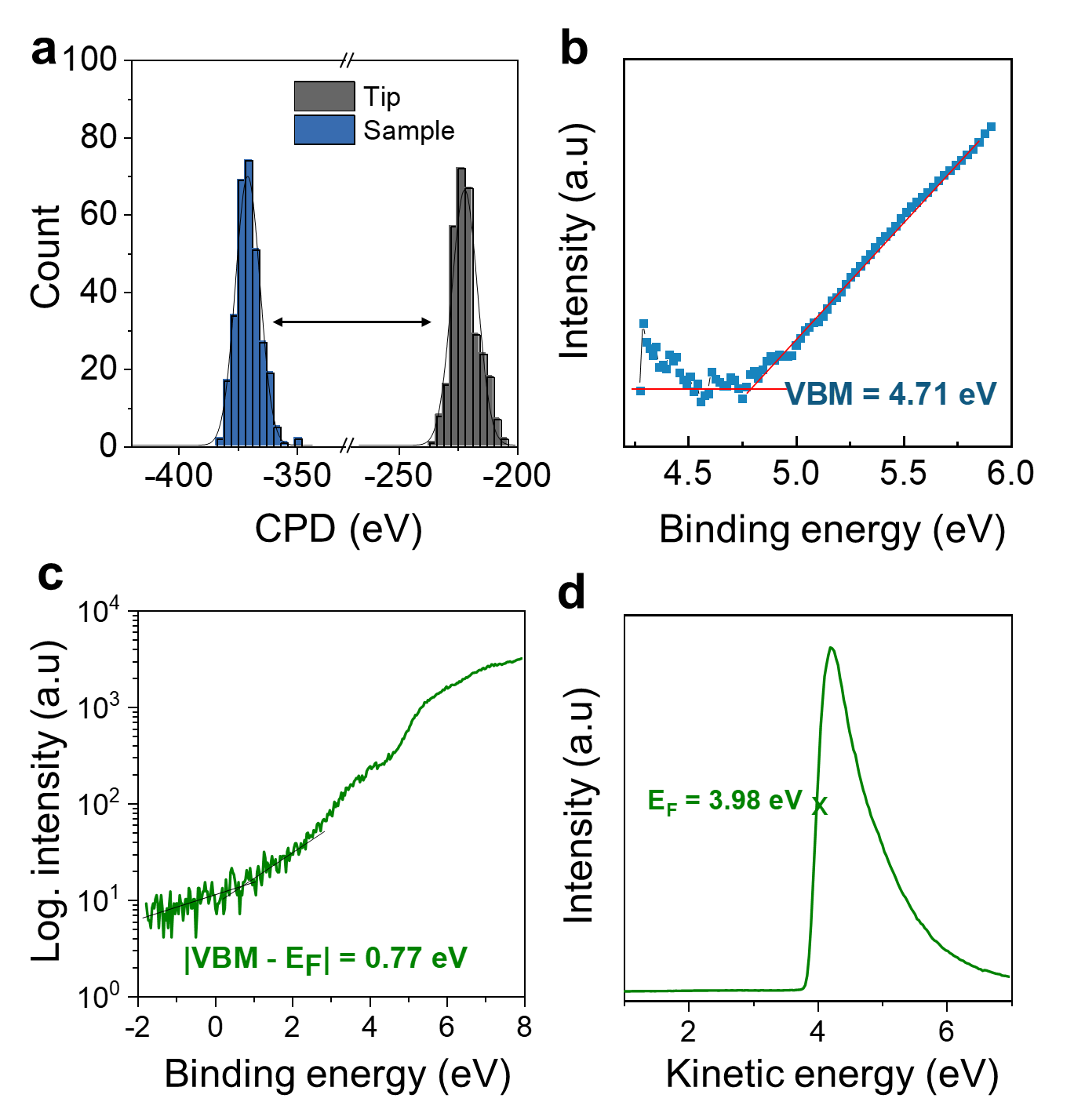


**Figure S8. Energy level of Cd_3_P_2_ films under ambient and high vacuum.** (a) Kelvin probe (KP) measurement of the tip and Cd_3_P_2_ sample. WF of the tip is calibrated by highly oriented pyrolytic graphite (HOPG), WF is 4.48 eV. 300 points of contact potential difference (CPD) are summarized in histogram. WF of Cd_3_P_2_ films is the summation of contact potential difference and WF of tip, calculated of 4.36 eV. (b) Valance band maximum of films is obtained in APS spectra. (c) Valence band region plotted on a logarithmic scale extracted from UPS and (d) secondary electron cutoff region used to determine the work function of 3.98 eV.


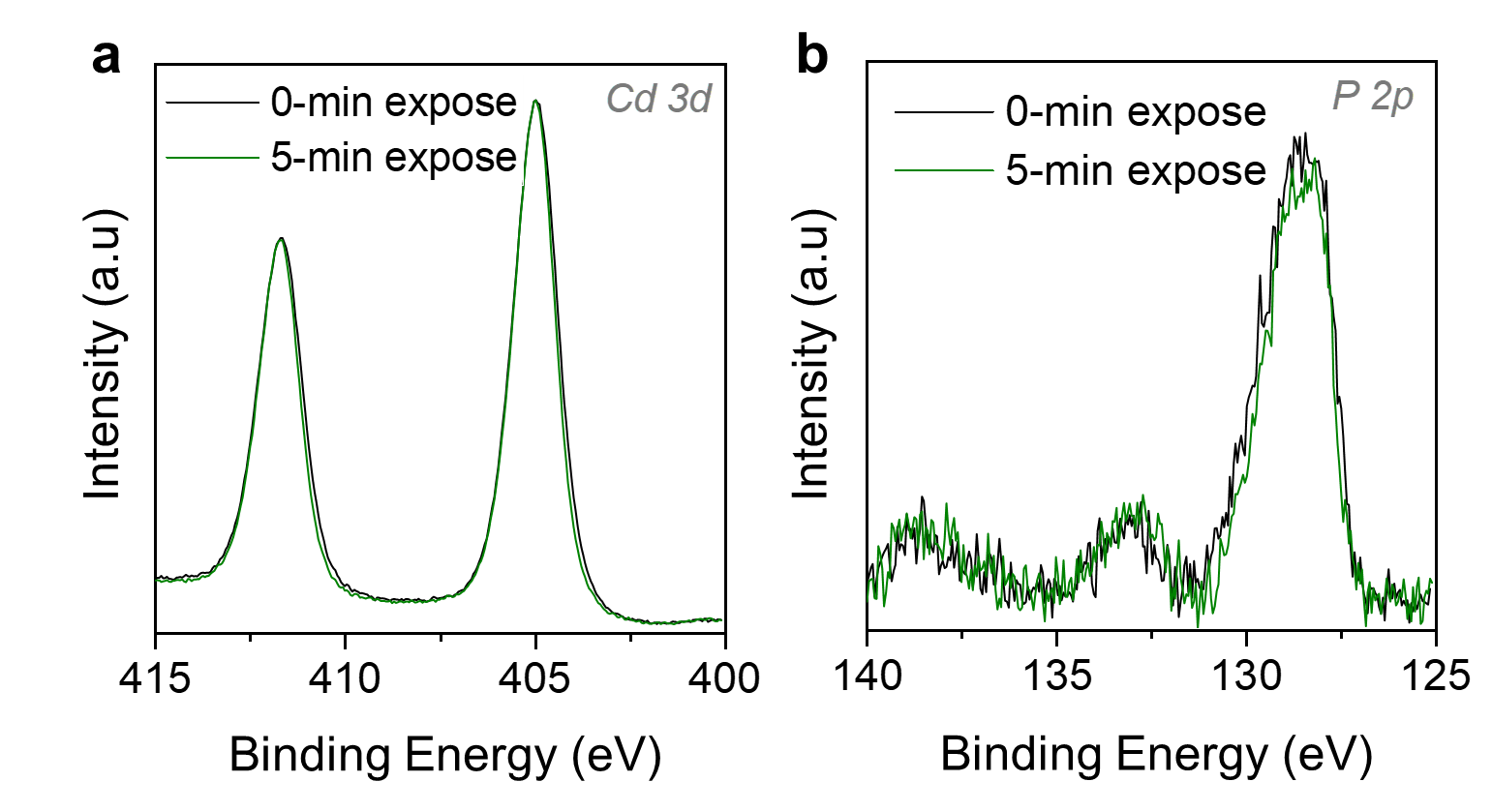


Figure S9. X-ray photoelectron spectroscopy (XPS) core-level spectra of Cd_3_P_2_–Cl CQD films. (a) Cd 3d and (b) P 2p spectra collected from the as-prepared film and after 5-minute air exposure.


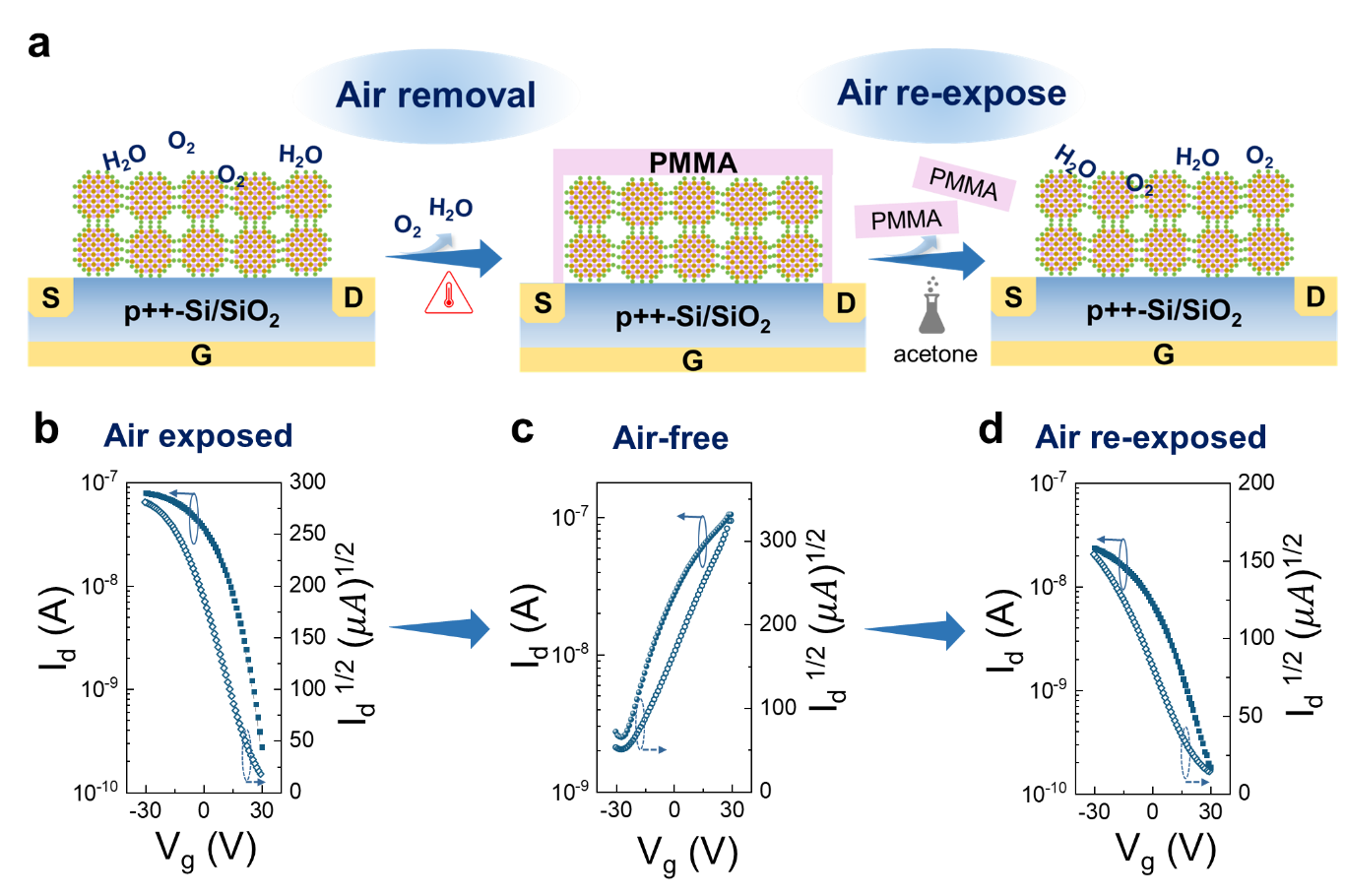


**Figure S10. Reversible carrier polarity control via encapsulation/decapsulation strategy.** (a) Schematic of the switching cycle. Air exposure facilitates ambient molecular adsorption, inducing the polarity switching. Annealing removes adsorbed species, followed by PMMA encapsulation to preserve the air-free state. PMMA is then removed by acetone to allow re-adsorption. (b) Air-exposed, unencapsulated device shows p-type behavior. (c) Post-annealing and PMMA encapsulation convert to n-type behavior, confirming air removal. (d) Decapsulation via acetone reinstates p-type polarity.


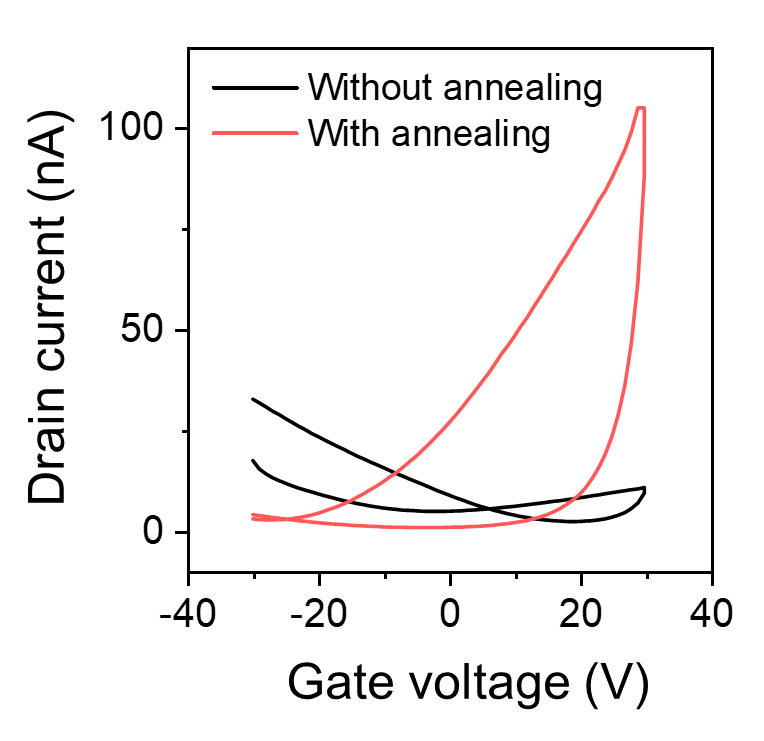


**Figure S11.** Transfer characteristics of Cd_3_P_2_ QD FETs without (black) and with (red) mild thermal annealing after air exposure following by PMMA encapsulation.


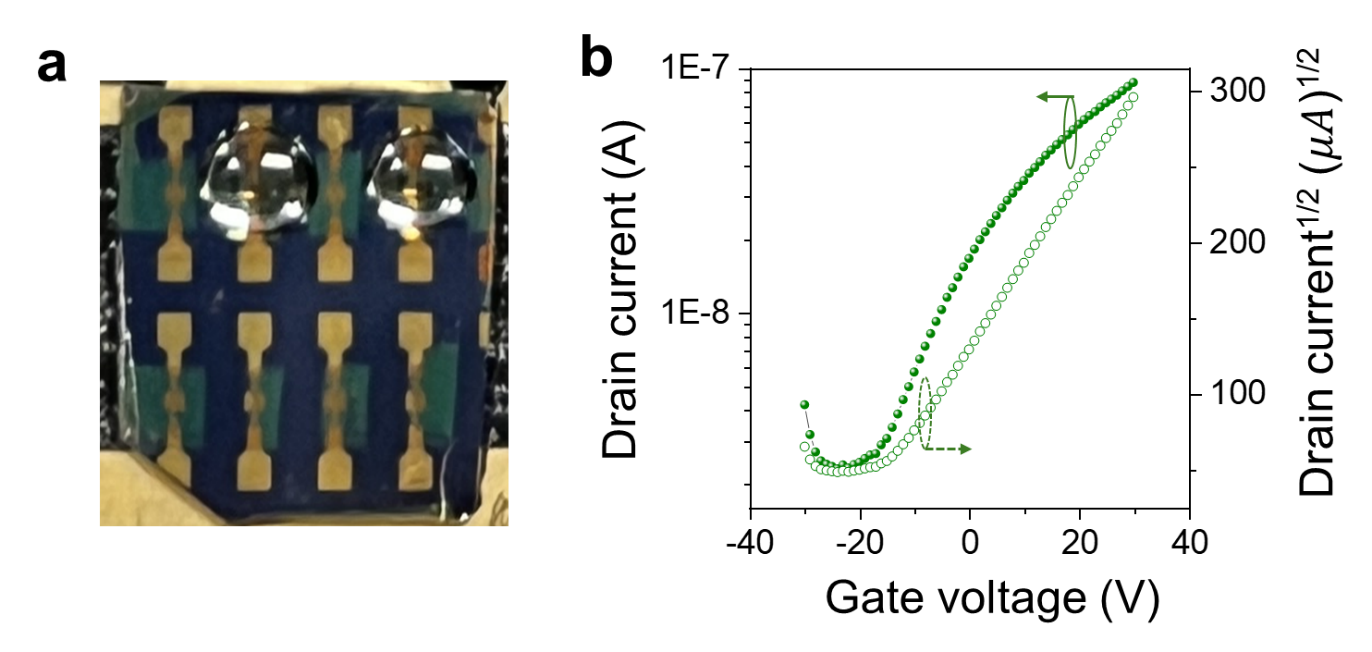


**Figure S12. UV-curing resin as an encapsulation layer.** (a) Photograph of a Cd_3_P_2_ CQD FET device encapsulated with UV-curing resin. (b) Transfer characteristics of the UV-resin-encapsulated device, exhibiting n-type conduction.


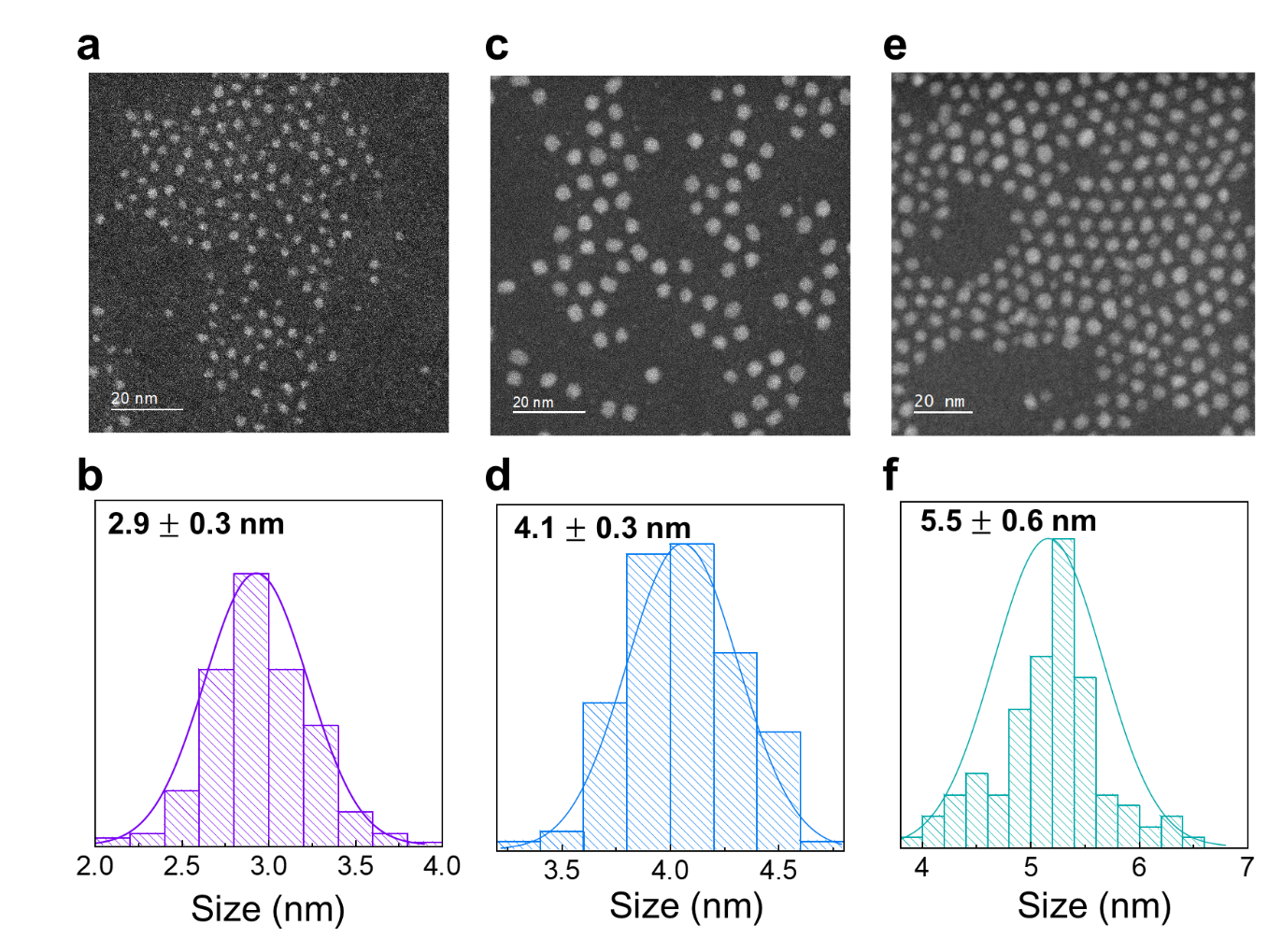


**Figure S13. Transmission electron microscopy (TEM) images and corresponding distribution histograms of colloidal Cd_3_P_2_ QDs at various size.** (a, b) 2.9 nm, (c, d) 4.1 nm, and (e, f) 5.5 nm, average diameters were obtained from TEM analysis of over 100 particles per sample.


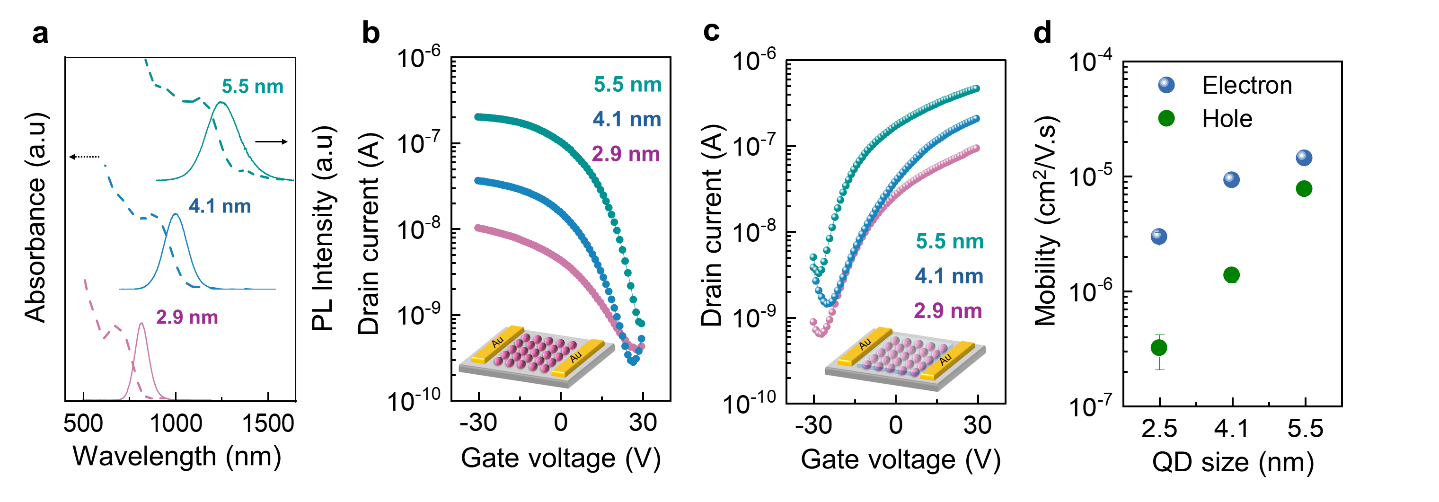


**Figure S14. Size-dependent transport properties of Cd_3_P_2_ QD films.** (a) Absorption (dashed line) and PL (solid line) spectra of QDs with average diameters of 2.9, 4.1, and 5.5 nm. (b) Transfer characteristics of air-exposed FETs for different QD sizes, measured under Vds = −30V. (c) Transfer characteristics of PMMA-encapsulated devices, measured under Vds = 30V. (d) Extracted size-dependent electron and hole mobilities.


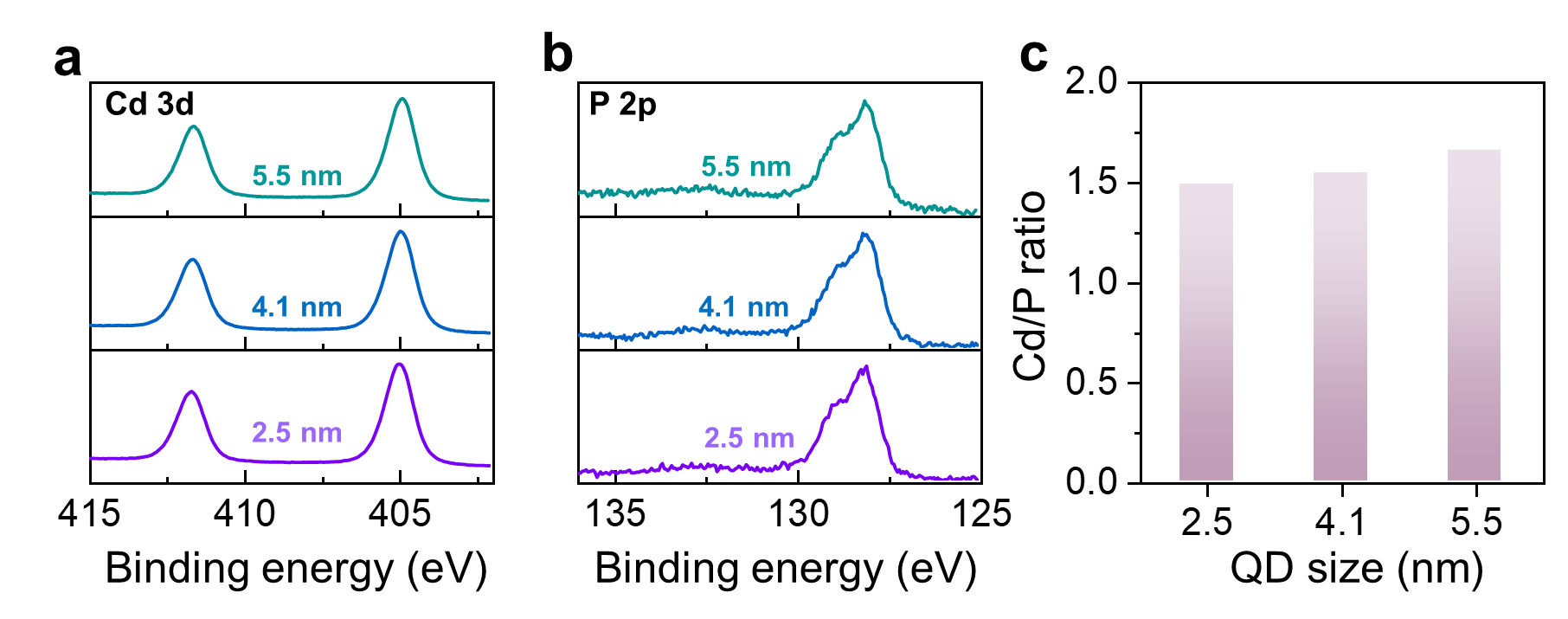


**Figure S15. X-ray photoelectron spectroscopy (XPS) analysis of Cd_3_P_2_** **CQDs with different mean sizes.** (a) Cd 3d and (b) P 2p core-level spectra for QDs with diameters of 2.5, 4.1, and 5.5 nm, respectively. (c) Size-dependent Cd/P atomic ratios extracted from XPS, showing a consistently Cd-rich composition across all sizes.


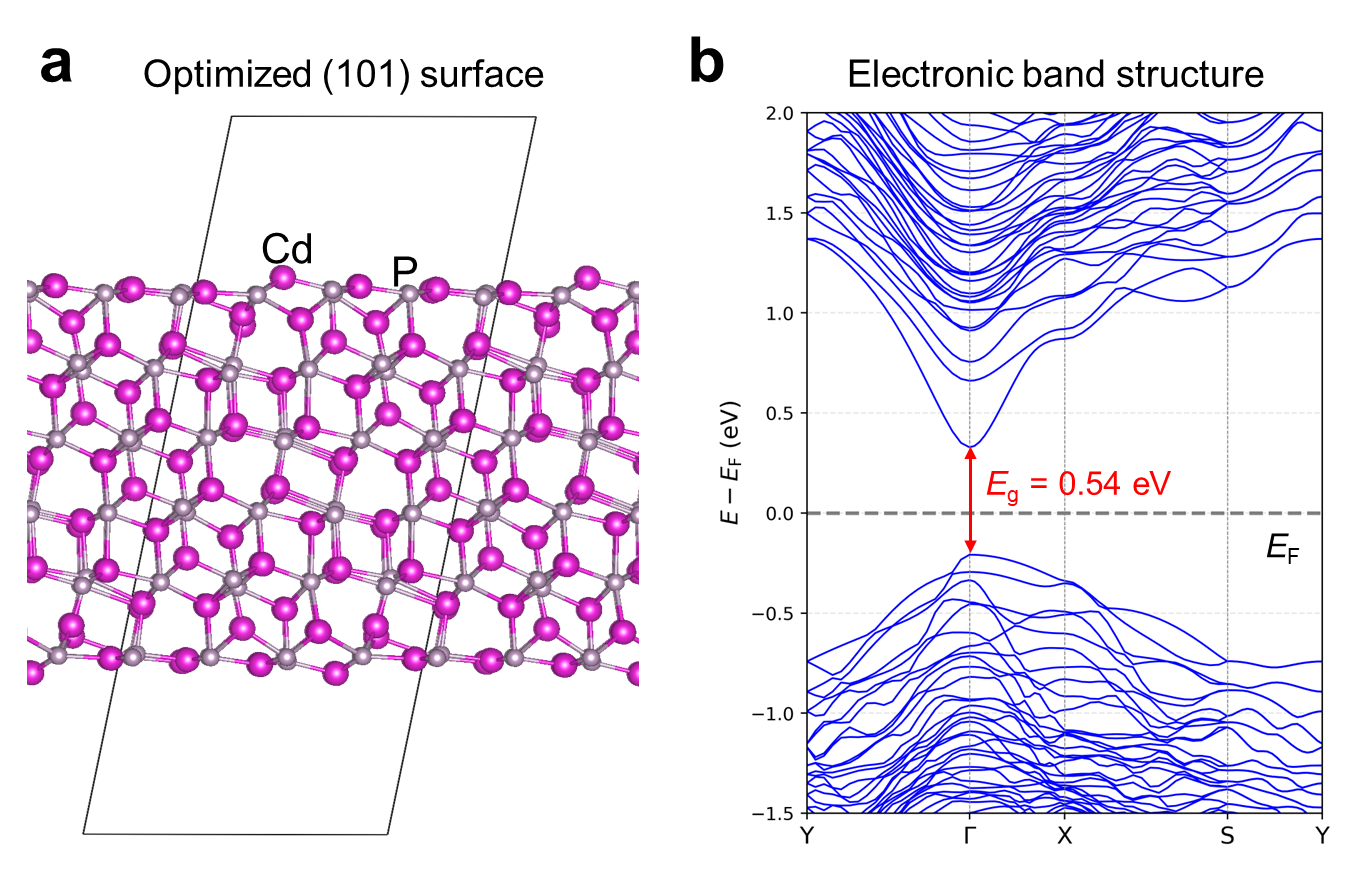


**Figure S16. Atomic and electronic band structure of the Cd_3_P_2_ (101) surface.** (a) Side view of ball-and-stick model of the bare Cd_3_P_2_ (101) surface. (b) Electronic band structure of this surface, which exhibits a direct band gap at the Γ-point. The Fermi level is indicated by the gray line.


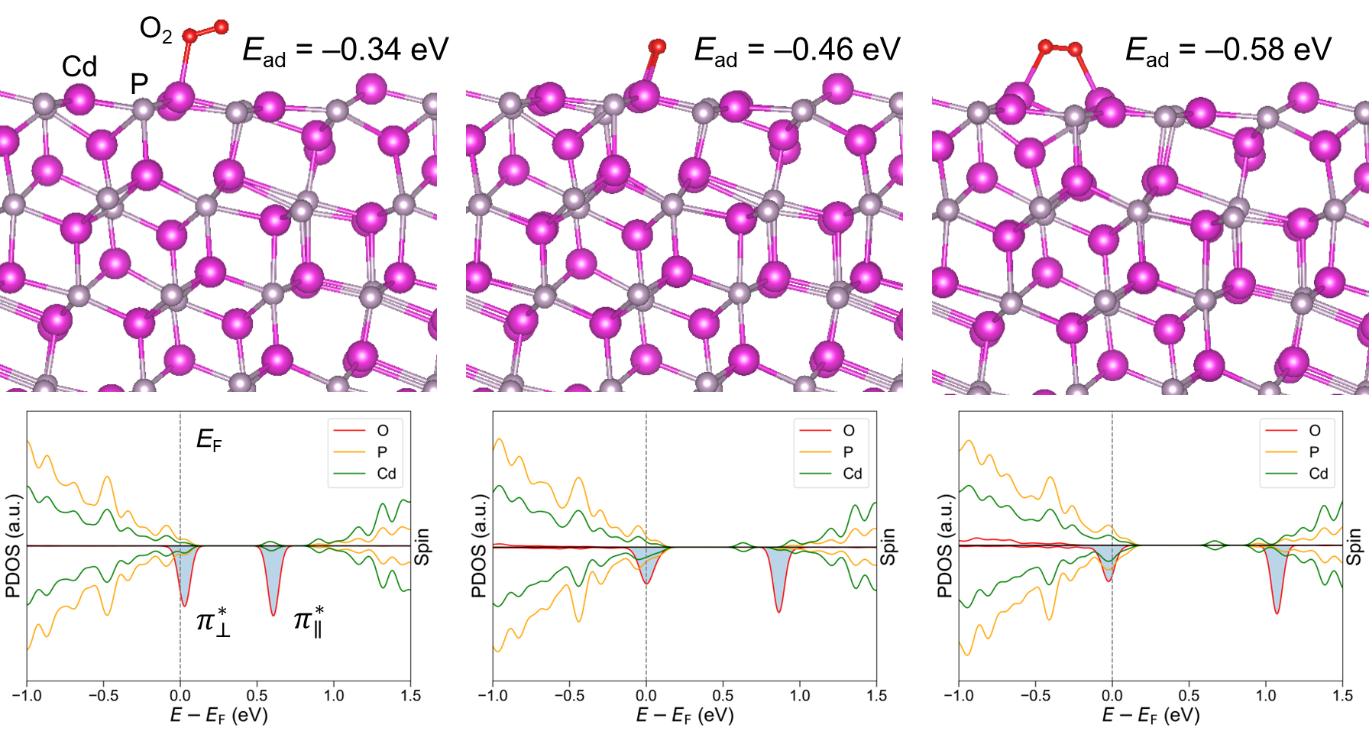


**Figure S17. Adsorption configurations of O_2_ on the Cd_3_P_2_ (101) surface and the corresponding spin-polarized partial density of states (PDOS).** Top panels: optimized atomic structures with adsorption energies calculated as –0.34, –0.46, and –0.58 eV. Bottom panels: spin-polarized PDOS for Cd, P, and O atoms, with the Fermi level indicated by the dashed gray line. The degree of occupation of the $\pi_{\perp}^{*}$ orbitals varies with adsorption energy, indicating that stronger binding promotes electron transfer from the surface to molecular oxygen.


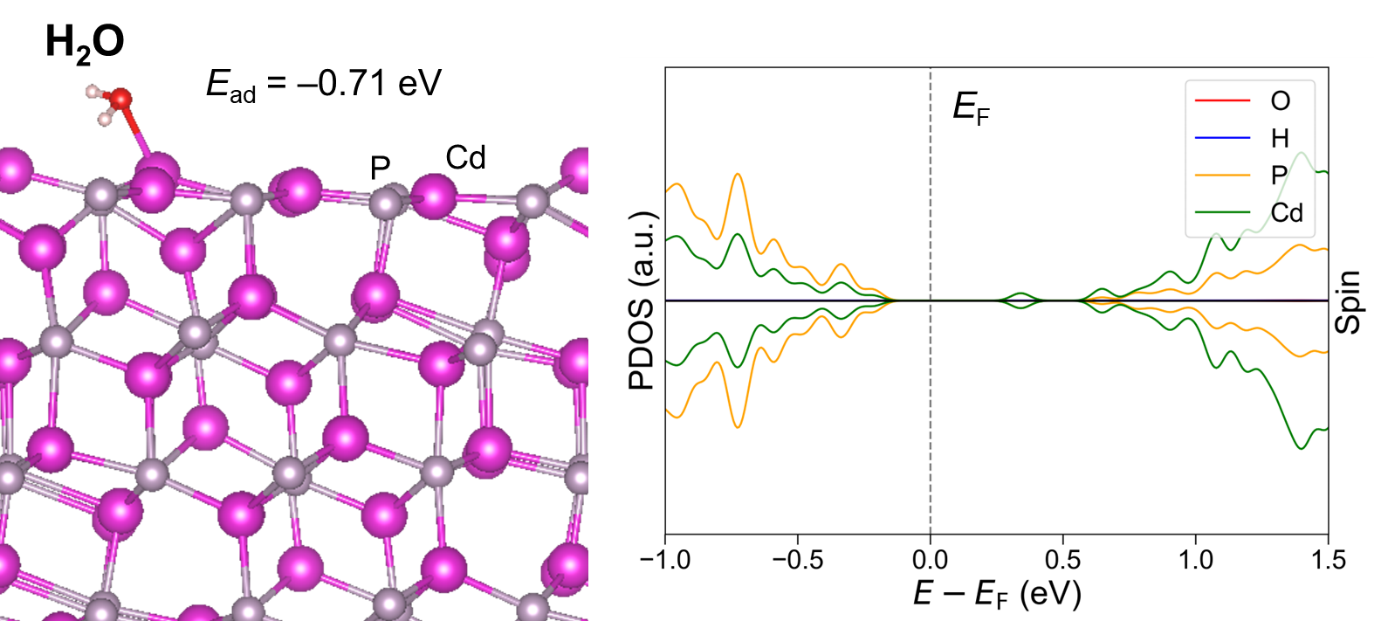


**Figure S18. Adsorption configuration of H_2_O on the Cd_3_P_2_ (101) surface and the corresponding spin-polarized partial density of states (PDOS).** Left panel: optimized atomic structure with a calculated adsorption energy of –0.71 eV. Right panel: spin-polarized PDOS for Cd, P, O, and H atoms, with the Fermi level indicated by the dashed gray line.


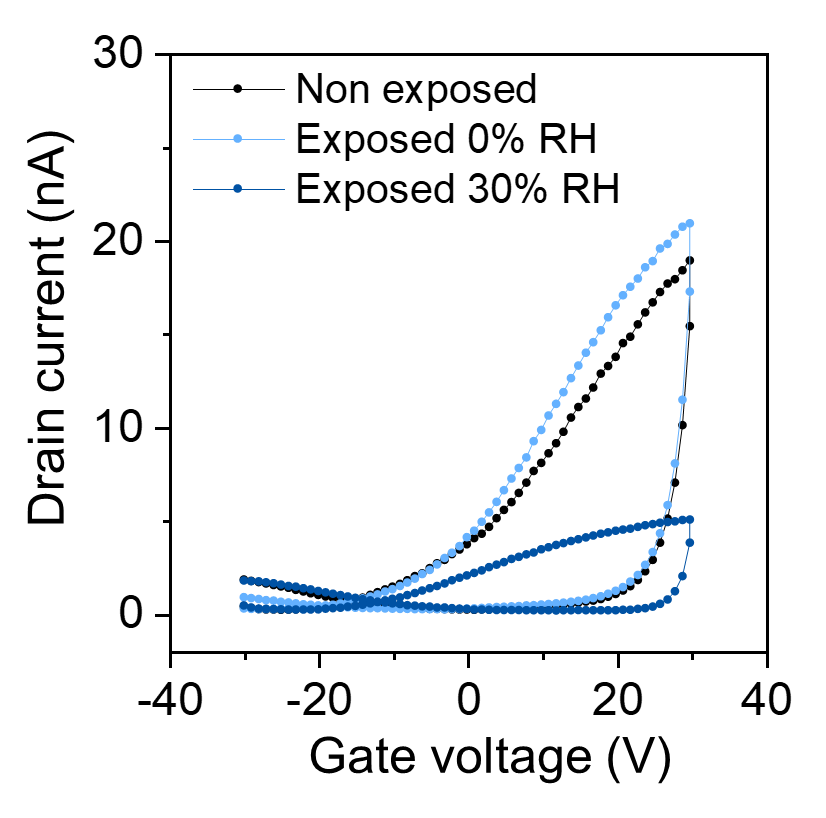


**Figure S19. Effect of humidity on the polarity switching behavior of Cd_3_P_2_ CQD FETs.** Transfer characteristics measured for devices without ambient exposure (non-exposed) and after controlled exposure under dry conditions (0% RH, ambient O_2_) and humid conditions (30% RH, ambient O_2_).


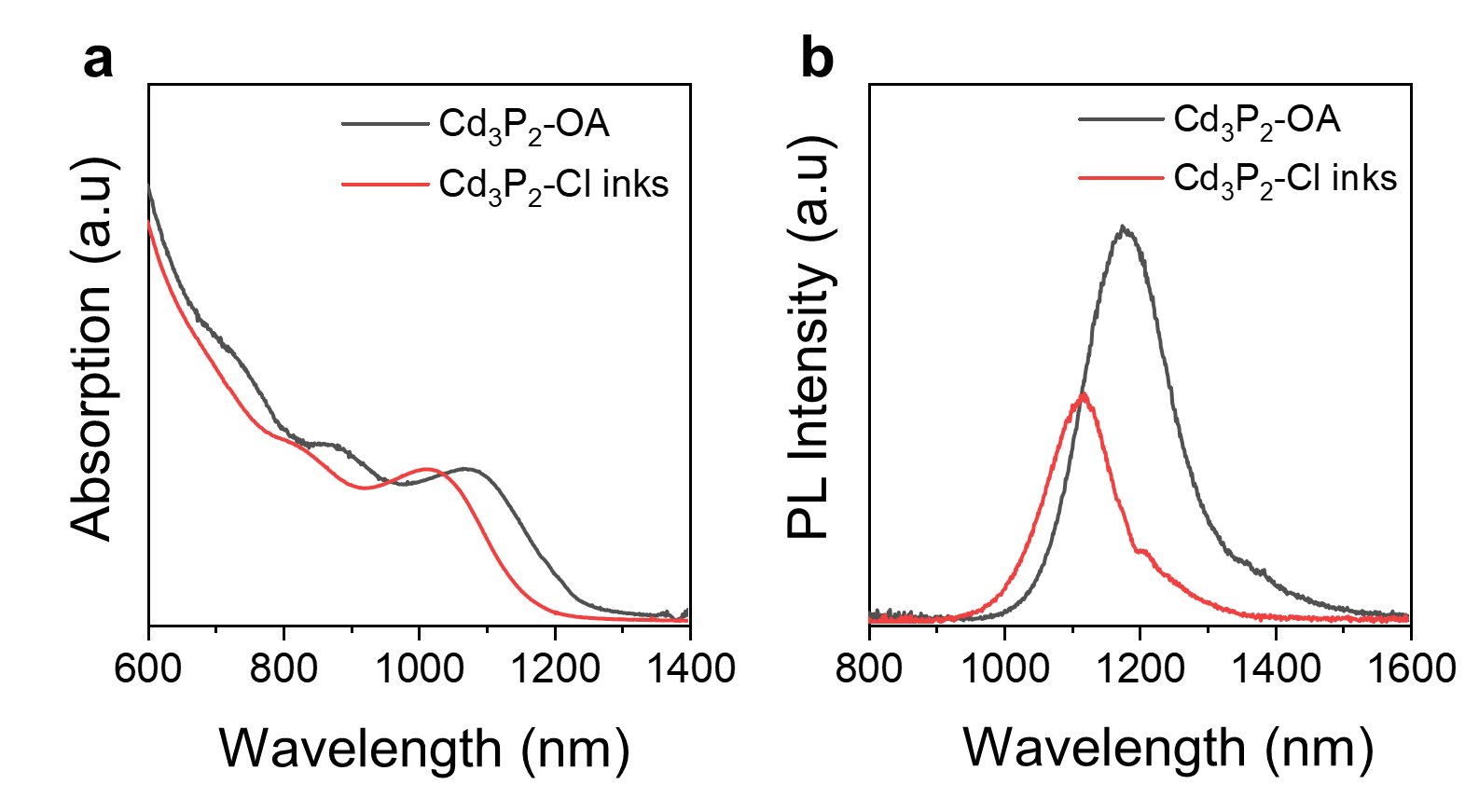


**Figure S20. Optical properties of Cd_3_P_2_ CQDs solution before and after ligand exchange**. (a) Absorption spectra of Cd_3_P_2_ films capped with native oleate ligands (Cd_3_P_2_ –OA) and after chloride-based ink processing (Cd_3_P_2_–Cl inks). (b) Corresponding photoluminescence (PL) spectra showing a blue-shifted and broadened emission for Cd_3_P_2_ –Cl inks relative to Cd_3_P_2_ –OA.

**Figure S21.** Detectivity of Cd_3_P_2_ CQD photodiodes at –0.5V.


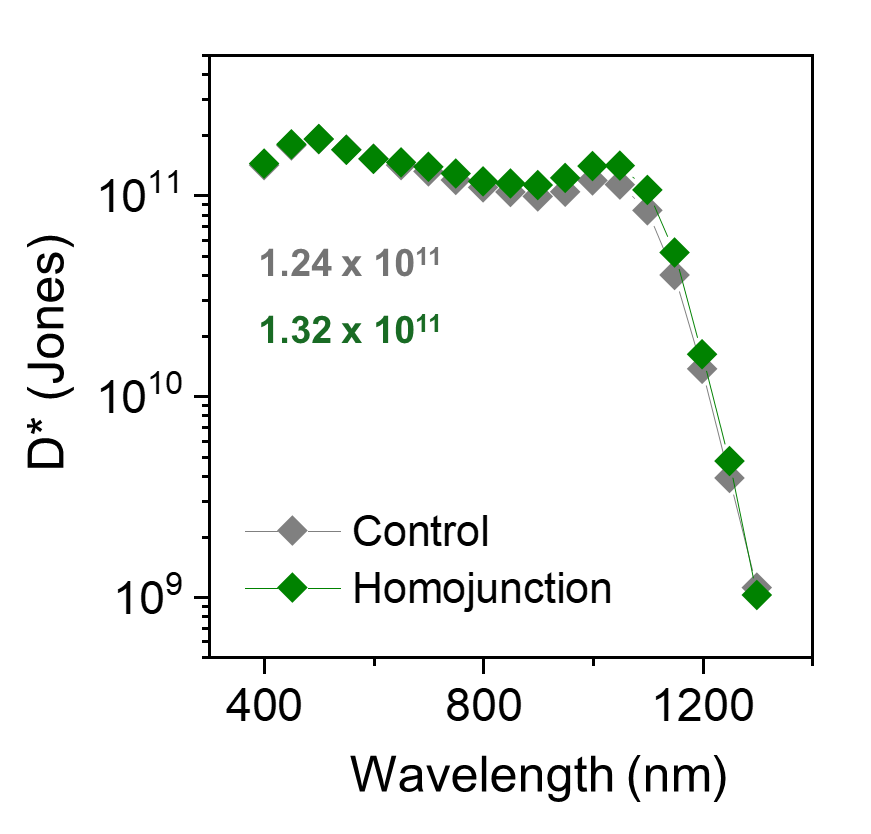


Table S1. Calculated surface energy of the bare surfaces of Cd_3_P_2_ (unit: meV Å^−2^)

| **Surface** | **(001)** | **(100)** | **(110)** | **(101)** | **(111)** |
| --- | --- | --- | --- | --- | --- |
| **Surface energy (𝛾)** | 64.50 | 40.17 | 56.07 | 27.56 | 43.22 |

Table S2. Comparison of CQD-based photodetections

| **Material** | **Wavelength**  **(nm)** | **Jsc**  **(mA/cm^2^)** | **EQE**  **(%)** | | **Rise/fall time**  **(ns)** | **D***  **(Jones)** | **Bias** | **Ref.** |
| --- | --- | --- | --- | --- | --- | --- | --- | --- |
| In(P)As | 1140 | - | | 5 | - | 1.1 × 10^10^ | -4 | ^[1]^ |
| InAs | 1020 | - | | 12.6 | 13500/9000 | 1.6 × 10^10^ | -0.5 | ^[2]^ |
| InAs | 1100 | - | | 40 | -/10 | 5.0 × 10^11^ | 0 | ^[3]^ |
| InAs | 1200 | 12.7 | | 38 | - | - | -0.5 | ^[4]^ |
| InAs | 1400 | - | | <1 | 1600/1500 | 1.0 × 10^9^ | 0 | ^[5]^ |
| InAs | 1500 | - | | 2.5 | -/1000 | 1.2 × 10^10^ | 1 | ^[6]^ |
| InSb | 1200 | - | | 2.7 | -/70 | 4.4 × 10^11^ | 0 | ^[7]^ |
| InSb | 1400 | - | | 3.4 | -/5600 | 1.4 × 10^11^ | 0 | ^[8]^ |
| Ag_2_Te | 1450 | - | | 20 | 25/- | 1.0 × 10^11^ | 0 | ^[9]^ |
| Ag_2_Te | 1500 | - | | 20 | 1000/4500 | 3.0 × 10^12^ | 0 | ^[10]^ |
| Ag_2_Te | 1550 | - | | 7 | -/38 | 1.1 × 10^11^ | 0 | ^[11]^ |
| Ag_2_Te | 1450 |  | | 30 | 25/- |  | -0.5 | ^[12]^ |
| **Cd_3_P_2_** | **1100** | **18** | | **24** | **23/250** | **1.3 × 10^11^** | **0** | **This work** |

**References**

[1] J. R. Manders, T.-H. Lai, Y. An, W. Xu, J. Lee, D. Y. Kim, G. Bosman, F. So, *Advanced Functional Materials* **2014**, *24*, 7205.

[2] B. K. Jung, H. Yoo, B. Seo, H. J. Choi, Y. K. Choi, T. H. Kim, N. Oh, S. Y. Kim, S. Kim, Y. Lee, J. W. Shim, H. Y. Park, G. W. Hwang, T. N. Ng, S. J. Oh, *ACS Energy Lett.* **2024**, *9*, 504.

[3] P. Xia, S. Wang, Y. Chen, A. Gulsaran, Y. Zhang, M. Vafaie, M. Imran, A. M. Najarian, Y. Liu, H. Ban, L. K. Sagar, M. Yavuz, E. H. Sargent, *Advanced Materials* **2025**, *37*, 2419020.

[4] Y. Park, J. Kim, M. Jeong, D. Shin, J. Jung, H. Kim, H. Jeong, H. Kim, Y.-H. Kim, S. Jeong, *Advanced Energy Materials* **2025**, *15*, 2404141.

[5] J. Leemans, V. Pejović, E. Georgitzikis, M. Minjauw, A. B. Siddik, Y.-H. Deng, Y. Kuang, G. Roelkens, C. Detavernier, I. Lieberman, P. E. Malinowski, D. Cheyns, Z. Hens, *Advanced Science* **2022**, *9*, 2200844.

[6] T. Sheikh, W. J. Mir, A. Alofi, M. Skoroterski, R. Zhou, S. Nematulloev, M. N. Hedhili, M. B. Hassine, M. S. Khan, K. E. Yorov, B. E. Hasanov, H. Liao, Y. Yang, A. Shamim, M. Abulikemu, O. F. Mohammed, O. M. Bakr, *J. Am. Chem. Soc.* **2024**, *146*, 29094.

[7] L. Peng, Y. Wang, Y. Ren, Z. Wang, P. Cao, G. Konstantatos, *ACS Nano* **2024**, *18*, 5113.

[8] Y. Zhang, P. Xia, B. Rehl, D. H. Parmar, D. Choi, M. Imran, Y. Chen, Y. Liu, M. Vafaie, C. Li, O. Atan, J. M. Pina, W. Paritmongkol, L. Levina, O. Voznyy, S. Hoogland, E. H. Sargent, *Angewandte Chemie* **2024**, *136*, e202316733.

[9] Y. Wang, H. Wu, C. Rodà, L. Peng, N. Taghipour, M. Dosil, G. Konstantatos, *Advanced Materials* **2025**, *37*, 2500977.

[10] Y. Wang, L. Peng, J. Schreier, Y. Bi, A. Black, A. Malla, S. Goossens, G. Konstantatos, *Nat. Photon.* **2024**, *18*, 236.

[11] J. A. Lee, I. S. Lee, D. Kang, N. Kim, J. Kim, S.-W. Baek, Y. Kim, *Small* **2025**, *21*, 2412420.

[12] Y. Wang, H. Wu, C. Rodà, L. Peng, N. Taghipour, M. Dosil, G. Konstantatos, *Advanced Materials* **2025**, *37*, 2500977.
